# Sustainable resource optimization for tomato cultivation in a rooftop greenhouse: an 8-year case study

**DOI:** 10.1007/s13593-026-01118-6

**Published:** 2026-06-23

**Authors:** Guido Evangelista, Gara Villalba, Francesco Orsini, Joan Muñoz-Liesa, Verónica Arcas-Pilz, Xavier Gabarrell

**Affiliations:** 1 Sostenipra Research Group (2021 SGR 00734), Unitat de excelencia Maria de Maetzu CEX2024001506-M, Institut de Ciència i Tecnologia Ambientals (ICTA-UAB), Universitat Autònoma de Barcelona, Barcelona, Spain; 2 Department of Chemical, Biological and Environmental Engineering, Universitat Autonoma de Barcelona (UAB), Campus UAB, 08193 Bellaterra, Barcelona, Spain; 3 DISTAL—Department of Agricultural and Food Sciences, Alma Mater Studiorum, University of Bologna, Bologna, 40127 Italy; 4 Department of Sustainable Development, Environmental Science and Engineering, KTH Royal Institute of Technology, Teknikringen 10B, Stockholm, 114 28 Sweden; 5 Life Cycle Assessment Research Group, Agroscope, Reckenholzstrasse 191, Zurich, 8046 Switzerland

**Keywords:** Rooftop greenhouse, Tomato, Hydroponics, Urban agriculture, Circular economy, Life cycle assessment

## Abstract

Tomato is one of the most popular and consumed crops worldwide and its cultivation via hydroponic techniques is widespread. In this study, we evaluated the performance of an evolving management strategy through an iterative system design over 8 years in an integrated-rooftop greenhouse in Barcelona. Yield, solar radiation, water and nutrient consumption, and environmental impacts through Life Cycle Assessment were analysed. We hypothesized that improving irrigation management, partially substituting mineral fertilizers with recovered nutrients, and ensuring optimal solar radiation would enhance tomato productivity while reducing the environmental impact. The highest productivity was obtained in 2023 (49 g plant^−1^ day^−1^), in a cycle using 100 g plant^−1^ of struvite and in a cycle using mineral fertilization. The replacement of deteriorated polycarbonate sheets in 2023 increased greenhouse solar transmissivity and led to the highest productivity. Life Cycle Assessment impacts were mainly driven by crop yields, growing system characteristics, and energy required to power artificial lighting or nebulization systems, among other inputs. The lowest environmental impacts were obtained in the 2017 cycle (0.54 kg CO_2_ eq.) followed by the 2023 cycle (0.94 kg CO_2_ eq.), in which the highest yields were produced thanks to optimal solar transmissivity, dynamic fertigation, and/or struvite. The highest environmental impacts (3.84–12.05 kg CO_2_ eq.) were achieved in cycles with the lowest productivity (0.9–3.3 kg m^−2^) and in cycles using artificial lighting in the greenhouse (3.71 kg CO_2_ eq.) or in an indoor environment (7.07 kg CO_2_ eq.). Our findings demonstrate that statistically higher yields and environmental sustainability can be achieved by adopting struvite-based fertilization and regularly replacing the greenhouse covering material to ensure maximum light transmissivity. This research provides valuable and novel insights into optimizing tomato cultivation in rooftop greenhouses, achieving constant yields, and reducing the environmental footprints thus contributing to sustainable food production.

**Supplementary Information:**

The online version contains supplementary material available at 10.1007/s13593-026-01118-6.

## Introduction

An increasing demand for fresh food is driving attention towards urban food production systems, which often rely on innovative cultivation methods to meet the needs of a growing population while minimizing environmental impacts. Urban agriculture is usually described as horticultural, agricultural, and farming activities that occur on small plots of land in and around urban centres (Ackerman et al. 2014). Urban agriculture has environmental benefits that include reducing city waste, improving energy circularity, urban biodiversity, and air quality, and overall reducing the environmental impacts of food production in cities through synergies with other urban systems (Orsini et al. 2013; Azunre et al. 2019; Royer et al. 2023; Arcas-Pilz et al. 2023; Arosemena Polo et al. 2024). Among urban agriculture models, rooftop farming has gained considerable attention as an efficient and environmentally friendly approach to grow within cities and close to the majority of consumers (Appolloni et al. 2021), taking advantage of unused spaces, reducing transportation distances and associated carbon emissions (Toboso-Chavero et al. 2023). Rooftop farming often involves hydroponic cultivation, in which plants are grown in a nutrient-rich solution rather than soil, to minimize the rooftop load (Muñoz-Liesa et al. 2020). Hydroponic systems offer reduced water and fertilizer requirements relative to conventional methods (Barbosa et al. 2015; Pomoni et al. 2023); however, they also incur substantial environmental impacts through substrate utilization (Toboso-Chavero et al. 2021) and, in rooftop farming, infrastructure requirements (Sanyé-Mengual et al. 2015). Furthermore, the long-term environmental performance and crop yield can be compromised by the deterioration of greenhouse covering materials; for instance, Muñoz-Liesa et al. (2022a) reported an annual loss of 1.4% in solar transmissivity in these settings, which directly limits photosynthetic efficiency.

To reduce the environmental impact, rooftop hydroponic systems can be optimized by adopting strategies such as rainwater harvesting, leachate recirculation, and nutrient recovery from waste to replace mineral fertilizers (Arcas-Pilz et al. 2023). Several studies have demonstrated that adopting these strategies can produce comparable or even higher yields than those obtained with conventional techniques while simultaneously reducing the associated environmental impacts. Replacing mineral fertilizer with struvite (MgNH_4_PO_4_·6H_2_O), a salt recovered from wastewater treatment plants (WWTPs), resulted in higher yields for lettuce, pepper, and beans when directly placed in the substrate (Arcas-Pilz et al. 2021, 2022). Carreras-Sempere et al. (2021) applied struvite fertigation in a hydroponic system, which produced tomato yields and qualities similar to those of plants grown with conventional fertilizer. Additionally, research conducted by Rufí-Salís et al. (2020b), demonstrated how phosphorus demand for agriculture in the Barcelona region could be fully met by struvite produced by a single WWTP, showing how this technique could reduce dependency on this non-renewable resource. Recirculating the nutrient solution within the same system or using it for other crop cycles can reduce the environmental impacts related to fertilization (Bugbee 2004; Muñoz et al. 2017). In a study by Parada et al. (2021), the adoption of recirculating techniques in tomato cultivation resulted in similar yields and lowered water use and environmental impacts compared to an open system. According to research by Rufí-Salís et al. (2020a) on green beans, a closed-loop system can save 40% of irrigation water and between 35% and 54% of nutrients daily. However, these studies represent one-time experiments applied to a single crop using one specific resource-reduction strategy, which makes it difficult to compare them and determine which is the optimal strategy. To address this gap, our study takes a longitudinal and comparative approach across multiple crop cycles of tomato, which is rarely seen in the current literature on rooftop farming and/or urban agriculture.

The hypothesis of this study is that productivity and environmental performance are jointly shaped by interacting management mechanisms — including changes in nutrient solutions, water recirculation practices, and solar radiation constraints — and that these mechanisms generate measurable trade-offs that can be progressively optimized through an iterative system design. Rather than testing isolated factors under controlled experimental conditions, the study evaluates how successive adjustments in crop nutrition, irrigation management, and radiation conditions influence system-level outcomes within real operational constraints.

**Fig. 1:**
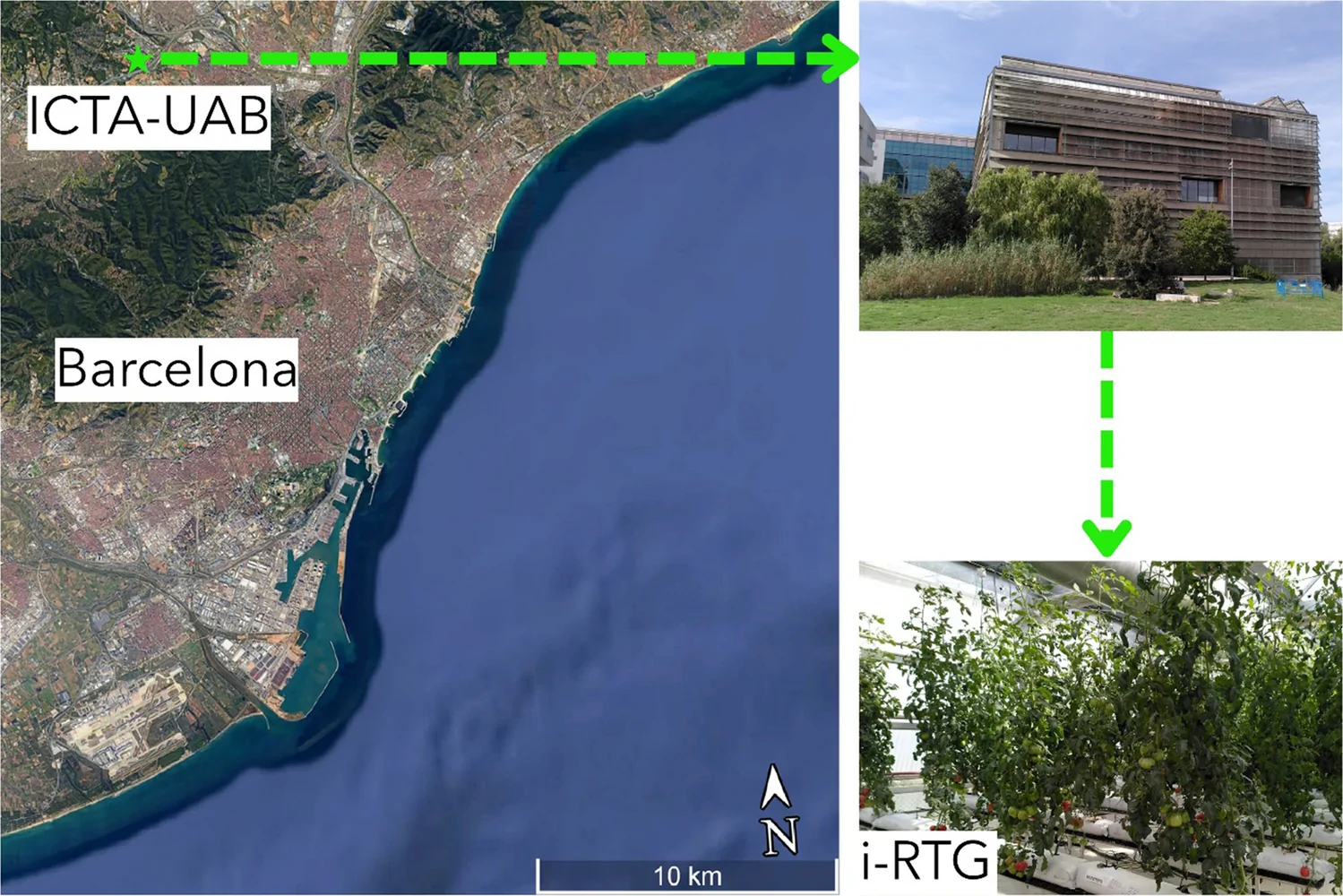
Description of the study site where most of the experiments took place. ICTA-UAB: Institute of Environmental Science and Technology of the Universitat Autònoma de Barcelona; i-RTG: integrate-rooftop greenhouse (Photo credit: Guido Evangelista).

This hypothesis was evaluated through the analysis of agronomic and environmental data from 11 tomato crop cycles cultivated using a hydroponic system over an eight-year period in an integrated-rooftop greenhouse (i-RTG) (Fig. 1). All cycles were conducted in the rooftop greenhouse, except for one performed in an indoor environment with artificial lighting. Across the study period, different tomato varieties were evaluated, while fertilization and irrigation strategies were progressively adjusted to reduce environmental impacts associated with the fertigation system. In parallel, radiation management was explored through two contrasting approaches: the addition of supplemental LED lighting and the replacement of greenhouse covering materials to improve solar transmissivity. Yield, water use, nutrient consumption, crop characteristics, and associated environmental impacts were assessed using Life Cycle Assessment (LCA) to identify trends, trade-offs, and potential areas for optimization.

The originality of this work lies in the integration of long-term agronomic monitoring with environmental assessment within an operational rooftop greenhouse, enabling the evaluation of how practice-driven management adjustments interact and change over time. The results of this study can offer practical guidance to farmers, agronomists, and stakeholders seeking to achieve high-yield tomato production while minimizing environmental impacts related to its inputs, particularly in urban or resource-constrained contexts.

## Materials and methods

In this study, a set of different strategies that aimed to improve irrigation, fertilization, and management practices were applied in the cultivation of 11 tomato crop cycles from 2015 to 2023. Most of the experiments were performed in a southeast-facing i-RTG at the Institute of Environmental Science and Technology (ICTA-UAB) building at the Universitat Autònoma de Barcelona (Catalunya, Spain), with an area of 125 m^2^. One experiment took place in the basement of the same building (see Appendix 1A for pictures of the location and extra information). Temperature, relative humidity, outdoor and indoor radiation were monitored daily with sensors (CS215 Campbell Scientific and L202 by Hukseflux). Passive ventilation was adjusted with an automated system by opening lateral and top windows to maintain temperature and humidity, and residual heat from the offices and laboratories in the building was used to passively warm the greenhouse during the coldest days, according to building ventilation requirements (Muñoz-Liesa et al. 2022a).

The tomato varieties used in this study with additional information on their characteristics are presented in Table 1.

**Table 1:** Main characteristics of tomato varieties used during the study.

Variety	Shape	Weight (g)	Provider
Arawak	Beefsteak	200–240	Syngenta
Tomawak	Beefsteak	200–240	Syngenta
Gigawak	Beefsteak	200–240	Syngenta
Siranzo	Truss	140–150	Rijk Swaan
Rosa de Cadiz	Beefsteak	300–600	Local provider
Montgrí	Flat and ribbed	200–400	Local provider

All crop cycles presented a single stem per plant, with plants grown in 40 L perlite bags and fertigated with a drip irrigation system using tap water or rainwater collected in a 100 m^3^ underground tank. The drip irrigation system had a flow rate of 2 L h^−1^, in which fertilizer was applied according to the phenological stage of the plants (Table 2). Nutrient concentration varied over the years and was based on experience gained during past experiments (Sanjuan-Delmás et al. 2019; Rufí-Salís et al. 2020a; Parada et al. 2021) as well as literature recommendations (Savvas et al. 2008, 2013). The substrate bags were placed initially on expanded polystyrene trays covered with a low-density polyethylene film, but from 2018 onwards, the polystyrene trays were replaced by aluminium trays with a height of 0.5 m. Both systems of trays were sloped to enable the collection of leachates with the purpose of analysing their nutrient content and potentially utilizing the solution for future crop cycles.

**Table 2:** Nutrient concentrations in the nutrient solution and number of fertilizations employed during the experiments. Contents of macronutrients and micronutrients are expressed in mM L^−1^ and µmol L^−1^, respectively. For experiments 22-EA, 22-ER, 23-EA and 23-EM, the contents of H_2_PO_4_^−^, NH_4_^+^ and Mg^2+^ are expressed in kg. *C* crop cycle with regular fertilization, *FW* Fall-Winter cycle, *RC* recirculation system, *RR* recirculation system and irrigation reduction, *IS* irrigation scheme, *IE* indoor environment, *L* supplemental lighting sector, *S* Siranzo cv., *G* Gigawak cv., *A* Arawak cv., *R* Rosa de Cadiz cv, *M* Montgrí cv., *E* crop cycle with struvite fertilization.

Experiment	Fertilizations	Days	NO_3_^-^	H_2_PO_4_^-^	SO_4_^2-^	Cl^-^	K^+^	Ca^2+^	Mg^2+^	NH_4_^+^	Fe	B	Mn	Zn	Cu	Mo
15-C	2	77	10	1	1.5	2	7	3	1.5	0	30.8	4.6	7.3	2.3	2.4	0.1
		87	11.5	1	2	2.5	7	4	2	0	30.8	4.6	7.3	2.3	2.4	0.1
15-FW	3	111	7	1	1.5	3	5	3	1.5	0	30.8	4.6	7.3	2.3	2.4	0.1
		21	7.5	1	1	3	5	3	1.25	0	30.8	4.6	7.3	2.3	2.4	0.1
		37	7.5	1	1	3	5	3	1.25	0	30.8	4.6	7.3	2.3	2.4	0.1
16-C	2	24	8.5	1	2	2	6	3.75	1	0	30.8	4.6	7.3	2.3	2.4	0.1
		122	9	1	1.5	2	6	3.5	1	0	30.8	4.6	7.3	2.3	2.4	0.1
17-C	6	8	5	1	1.25	2	4.5	2.5	0.5	0	30.8	4.6	7.3	2.3	2.4	0.1
		29	7.5	1	1.25	1.5	4.5	3	1	0	30.8	4.6	7.3	2.3	2.4	0.1
		11	8	1	2.5	2	7.5	3.3	1	0	30.8	4.6	7.3	2.3	2.4	0.1
		25	8.5	1	2.5	0.5	7	4.25	0.5	0	30.8	4.6	7.3	2.3	2.4	0.1
		41	10	1	2.5	0.75	8	4.75	0.5	0	30.8	4.6	7.3	2.3	2.4	0.1
		75	8.5	1	2.5	0.75	7.5	4.5	0.5	0	30.8	4.6	7.3	2.3	2.4	0.1
18-C	1	201	5	1	1.25	2	4.5	2.5	1	0	30.8	4.6	7.3	2.3	2.4	0.1
19-RC, 19-RR	1	200	5	1	1.25	2	4.5	2.5	0.5	0	30.8	4.6	7.3	2.3	2.4	0.1
20-C, 20-IS	1	157	5	1	1.25	2	4.5	2.5	0.5	0	30.8	4.6	7.3	2.3	2.4	0.1
20-IE	1	146	5	1	1.25	2	4.5	2.5	0.5	0	30.8	4.6	7.3	2.3	2.4	0.1
21-L, 21-S, 21-G	2	89	9	1.25	2	2.5	6.75	4.25	0.75	0	30.8	4.6	7.3	2.3	2.4	0.1
		38	8.5	1	2	2.5	5.5	4.25	1	0	30.8	4.6	7.3	2.3	2.4	0.1
22-CA, 22-CR	2	105	9	1.3	2.25	2.5	6.75	4.25	1	0	30.8	4.6	7.3	2.3	2.4	0.1
		24	9	1.5	2.25	2.5	7	4.25	1	0	30.8	4.6	7.3	2.3	2.4	0.1
22-EA, 22-ER	2	105	5	2.41	1.25	2	3.5	2.75	0.61	0.45	30.8	4.6	7.3	2.3	2.4	0.1
		24	7.5		0	2.5	0	3.25			30.8	4.6	7.3	2.3	2.4	0.1
23-CA, 23-CM	3	41	10	1	2.5	3	6.5	4.25	2	0	23.6	20	10	6	0.8	0.5
		26	9	1.25	2.9	3	7	4.25	1.75	0	23.6	20	10	6	0.8	0.5
		78	8.5	1.25	2.9	3.5	7	4.5	1.25	0.5	23.6	22	10	6	1	1
23-EA, 23-EM	3	41	10	1.1	2.5	0	6.5	4.25	0.29	0.21	23.6	20	10	6	0.8	0.5
		26	9		3.25	0	7	4.25			23.6	20	10	6	0.8	0.5
		78	8		3.5	1	7	4.5			23.6	22	10	6	1	1

Additional details on the methodology of specific crop cycles can be found in the supplementary information (Appendix 1B).

### Yield analysis

Yield (g plant^−1^ day^−1^) across experiments that employed different plant densities and cycle lengths was calculated (Table 3), as shown in Eq. (1).

1 $$Yield=\frac{Grams\;of\;tomatoes\;produced\;(g)}{Plant\;\left(n\right)\times Days\;of\;the\;crop\;cycle\;(day)}$$

**Table 3:** Main characteristics, results and objectives of each tomato experiment for years 2015–2023 in the i-RTG. *WUE* water use efficiency, *A* Arawak cv., *T* Tomawak cv., *S* Siranzo cv., *G* Gigawak cv., *R* Rosa de Cadiz cv., *M* Montgrí cv., *C* crop cycle with regular fertilization, *FW* Fall-Winter cycle, *RC* recirculation system, *RR* recirculation system and irrigation reduction, *IS* irrigation scheme, *IE* indoor environment, *L* supplemental lighting sector, *E* crop cycle with struvite fertilization. Additional information on yield, water consumption, temperature, humidity and radiation can be found in the Appendix 2.

Experiment	Days	Variety	Plants	Yield (g plant^−1^ day^−1^)	WUE (g L^−1^)	Solar transmissivity (%)	Density (plant bag^−1^)
15-C	165	A	171	45.5	15.7	50.9	3
15-FW	171	T	171	12.9	8.4	46.8	3
16-C	148	A	171	35.1	20.9	48.6	3
17-C	188	A	171	45.2	22.9	47.2	3
18-C	196	A	171	32.1	13.8	41.5	3
19-RC	201	A	90	31.3	19.4	43.5	3
19-RR	201	A	81	30.8	17.05	43.5	3
20-C	158	A	90	31	22.3	40.9	3
20-IS	158	A	81	29.7	36.8	40.9	3
20-IE	146	A	18	24.7	30.3	-	3
21-L	128	S	60	26.6	16.6	39	3
21-S	128	S	30	18.8	11.9	39	3
21-G	128	G	81	30.5	17.9	39	3
22-CA	128	A	30	31.3	7.9	39.6	2
22-CR	128	R	20	14.1	3.6	39.6	2
22-EA	128	A	26	10.2	5.2	39.6	2
22-ER	128	R	18	3.9	2.1	39.6	2
23-CA	145	A	26	49	22.9	46.4	2
23-CM	145	M	30	35.9	16.5	46.4	2
23-EA	145	A	28	49	20.4	46.4	2
23-EM	145	M	30	36.6	14.6	46.4	2

Additionally, yield per square metre was calculated as shown in Eq. (2).

2 $$Yield=\frac{Kilos\;of\;tomatoes\;produced\;(Kg)}{Harvesting\;area\;(m^2)}$$

A one-way ANOVA followed by a Tukey post hoc test was conducted to assess significant differences among the yield values (*p* < 0.05), expressed in kg m^−2^, kg plant^−1^ and g plant^−1^ day^−1^. Significant differences of 22-CR and 22-ER were not analysed due to lack of data.

### Water balance

The water balance was calculated for each experiment and sector to determine water-use efficiency (WUE). WUE is the amount of biomass or fruit produced per unit of water used by a plant during a cycle (Hatfield and Dold 2019), and it may be used to compare between different production systems and cultivation methods (Table 2). In this study, WUE was calculated as the weight of harvested tomatoes (in grams) divided by the net water input (in litres), calculated as the total amount of water supplied to the crops minus the volume of recirculated solution, as shown in Eq. (3).

3 $$WUE=\frac{Grams\;of\;tomatoes\;produced\;(g)}{Total\;amount\;of\;water\;supplied\;to\;the\;crop\;\left(L\right)-Recirculated\;solution\;(L)}$$

### Nutrient balance

**Fig. 2:**
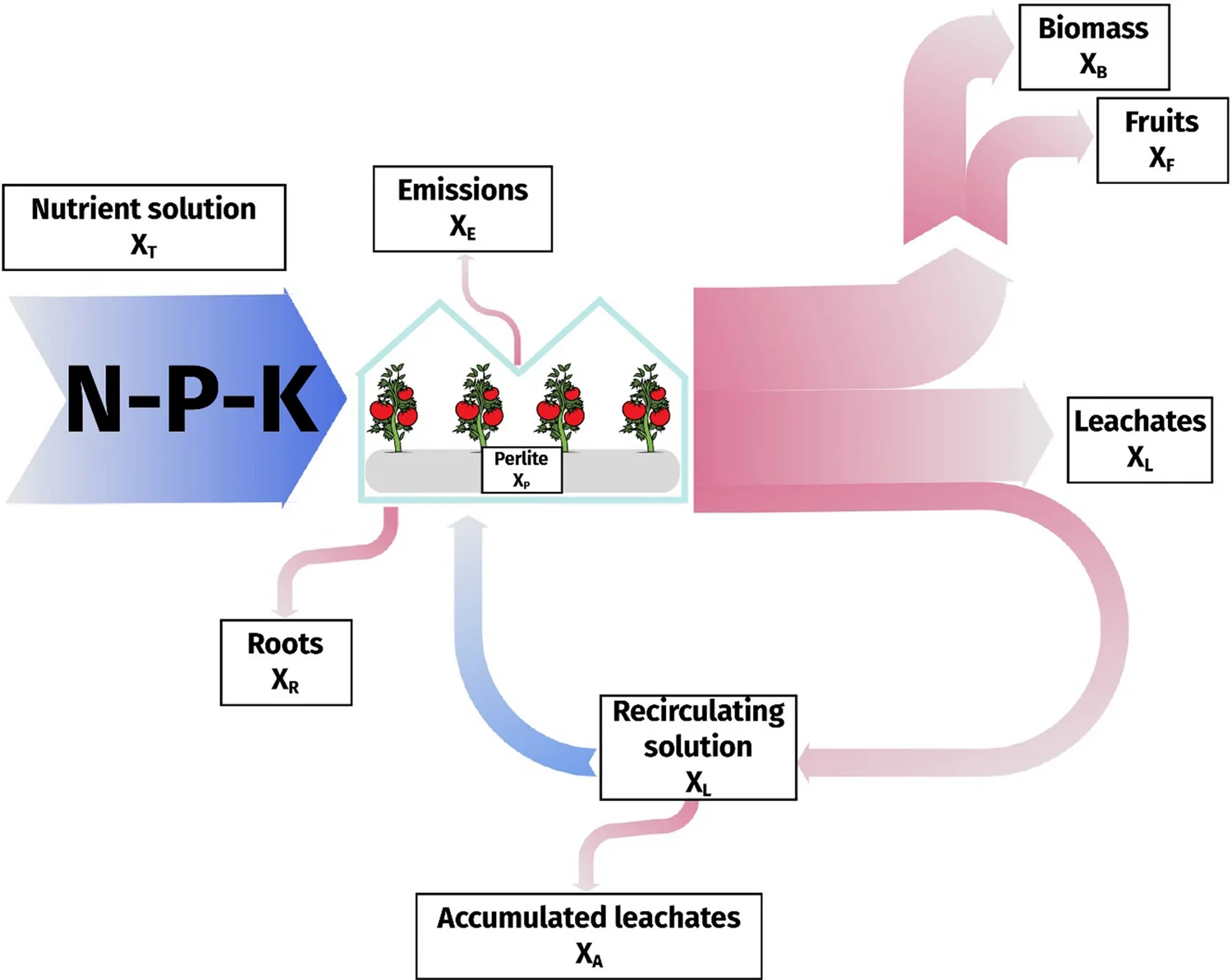
Flow diagram of N, P and K inputs and outputs in the open and closed hydroponics system of the integrated-rooftop greenhouse.

The nutrient balance was evaluated by quantifying the nitrogen, phosphorus, and potassium inputs via the irrigation system and outputs via plant biomass (leaves, stems, fruits, and roots) as well as potential nutrient loss through leaching and retention in the substrate, as shown in Fig. 2. This was done to understand the assimilation and allocation of nutrients in plant organs, losses through leaching, and residual nutrients remaining in the substrate. The data were used to calculate the emissions to water and air.

A total nutrient balance was calculated using Eq. (4), as illustrated in Fig. 2, where *X*_*T*_ represents the nutrients supplied by irrigation, *X*_*L*_ represents nutrients contained in the leachates, and *X*_*A*_ represents the nutrients accumulated in leachates and stored in a tank. These leachates were not used because of their high electrical conductivity values (~4 dSm^−1^). *X*_*B*_ and *X*_*F*_ represent the nutrients in plant biomass (leaves, stems, and fruits), whereas *X*_*P*_ and *X*_*R*_ represent the nutrients retained in the perlite and roots, respectively. Finally, *X*_*E*_ represents emissions to the atmosphere from nitrogen inputs (NH_3_, N_2_O, and NO_x_), which were calculated according to Montero et al. (2011) and the IPCC (De Klein et al. 2006).

A detailed methodology for the analysis of water, plant organs and perlite samples is given in Appendix 1 C, and the results are presented in Appendix 1D.

4 $${X}_{T}= {X}_{L}{+ X}_{A}+ {X}_{B}+ {X}_{F}+ {{X}_{P} {+ X}_{R}+X}_{E}$$

### Environmental and chemical parameters

The temperature, relative humidity, and indoor and outdoor radiation were continuously monitored (CS215, Campbell Scientific and Hukseflux LP02) and average values were recorded at 10-min intervals with a datalogger (CR3000, Campbell Scientific Inc.). For indoor and outdoor radiation, the values were integrated hourly to calculate the daily accumulated global radiation (in MJ m^−2^ day^−1^), and the overall transmissivity was obtained from the ratio of indoor to outdoor radiation, as described by Muñoz-Liesa et al. (2022b). Light use efficiency (LUE: g FW mol^−1^) was calculated as the ratio of the cumulative fresh weight of tomatoes and the cumulative amount of photosynthetically active radiation (PAR) received by the plants, which was measured by a sensor placed approximately 3 m from the ground (SKP215 Quantum sensor, Skye Instruments Ltd.).

The pH and electrical conductivity of the rainwater, irrigation water, and leachate were measured twice per week during the crop cycle.

### Life Cycle Assessment

#### Goal and scope

**Fig. 3:**
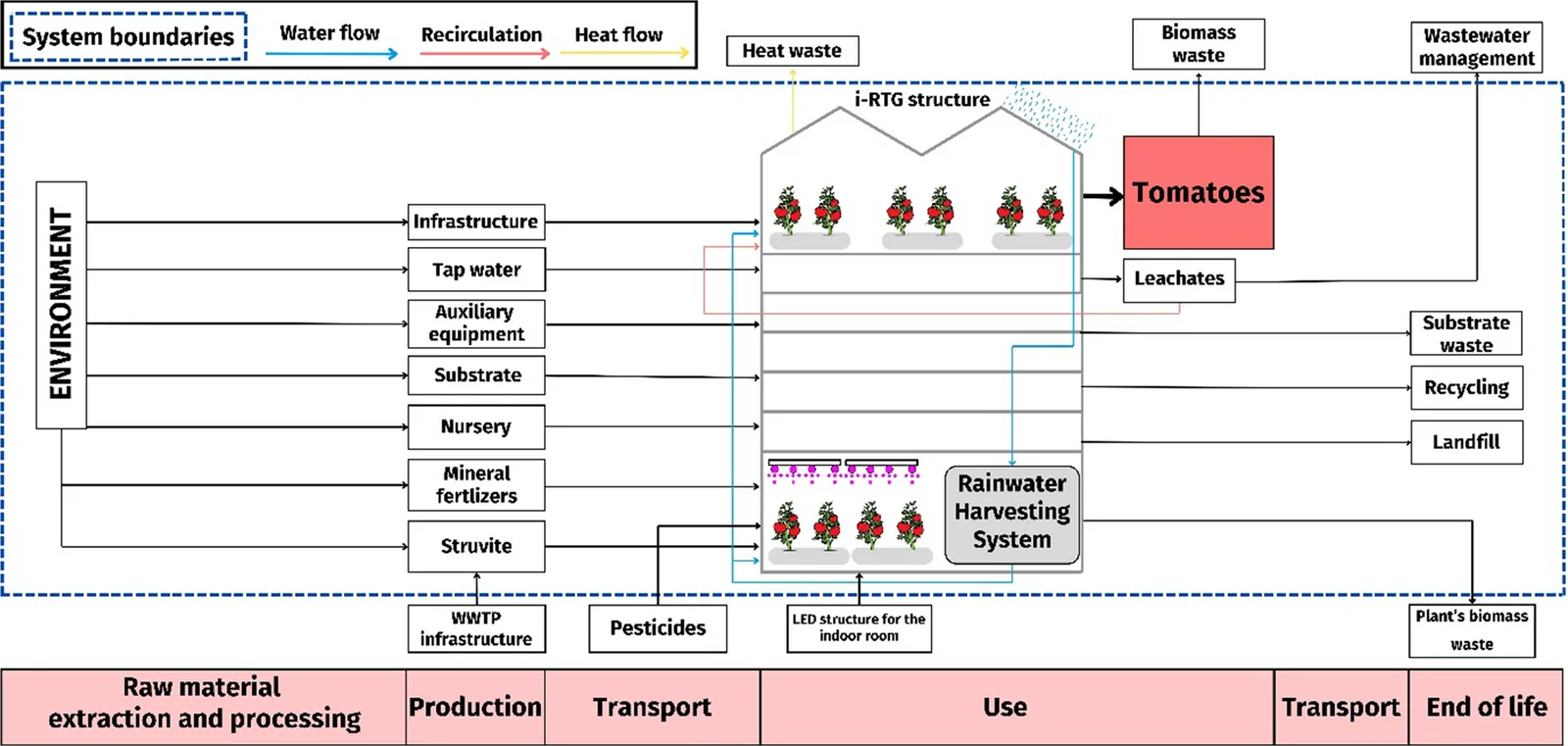
Scheme of system boundaries used for the case study.

Life Cycle Assessment (ISO 14040, 2006) was performed to assess the environmental impacts of the tomato experiments. Tomato crop cycles were compared by selecting 1 kg of tomato as the functional unit (FU), as it is commonly assessed in previous studies involving urban agricultural production (Rufí-Salís et al. 2020c; Parada et al. 2021; Farahani et al. 2019; Parajuli et al. 2021; Martin et al. 2023). The same system boundaries were applied to all 11 tomato experiments, from raw material extraction and the production of tomato nursery plants to their growth in the i-RTG (i.e. cradle-to-gate approach, Fig. 3).

#### Life cycle inventory

The inventory for the i-RTG was divided into four categories:

1. Auxiliary equipment: this category includes the headboard, tubes, pipes for distribution, water tanks, leachate trays, and all processes related to the transport, recycling, and end-of-life of these materials. Previous inventories were updated and adapted when needed for this study; for example, the nebulization system and the LED modules not previously been taken into account (Muñoz-Liesa et al. 2022b; Parada et al. 2021; Rufí-Salís et al. 2020a, 2020c; Sanjuan-Delmás et al. 2018). The processes included in this category were similar in every campaign, but a few additions were made throughout the years. For the allocation factor, the area occupied by the experiment was used to calculate the impacts.
2. Crop operation: this category includes the impacts of tap water use (for the irrigation and nebulization systems), fertiliser, substrate, emissions to water and air, and processes related to the transport, recycling, and disposal of these inputs. For emissions to water, no wastewater scenario was considered since there was no evidence that the discharged nutrients would be treated conventionally in a WWTP. The impacts related to the nursery stage of the plants (energy consumption and transport to the facility) were also included in this category. The impacts from the production of struvite were included in this category and were obtained according to Arcas-Pilz et al. (2022), without including the infrastructure of the WWTP in the system boundaries. For allocation, the area occupied by the experiment was used to calculate the impacts.
3. Rainwater harvesting system: this category includes the processes related to the construction of the system, storage and use of rainwater for crop cultivation, and the transport, recycling, and disposal of the materials. The processes included in this category were the same for every crop cycle. An allocation procedure based on the rainwater volume consumed in the i-RTG in relation to the harvested rainwater that was used to water other building plants was adopted to calculate the share of environmental impacts according to Sanjuan-Delmás et al. (2018).
4. Rooftop greenhouse structure: this category includes the processes related to the foundation and structure of the rooftop greenhouse and the transportation, operation, and end-of-life of these materials. The processes included in this category were the same for every crop cycle. This structure was retrieved from past inventories that were progressively updated (Sanyé-Mengual et al. 2015; Muñoz-Liesa et al. 2021). The allocated impact was based on the area occupied by each of the experiments (i.e., the proportion of area occupied by the 125 m^2^ of the i-RTG, including the area for greenhouse facilities).

For the experiment that was set in the indoor environment, the rainwater harvesting system and similar processes for auxiliary equipment and crop operation were employed. Impacts related to the construction of the building, the structure that supported the plants, and the energy needed to maintain stable environmental conditions were not considered in this experiment. The allocation impacts followed the same approach as previous assessments for this rooftop greenhouse (Muñoz-Liesa et al. 2022a).

Processes related to the production and use of pesticides and disposal of residual biomass were excluded from the system boundaries in the experiments because of relatively low impact and lack of data (Sanjuan-Delmás et al. 2018). Moreover, the passively and actively energy benefits that the i-RTG provide to the building acting as insulation layer have been excluded to simplify the analysis, since the focus here was to assess the relative environmental impacts of the different tomato campaigns (Muñoz-Liesa et al. 2022b). More details on the inventory can be found in the supplementary materials (Appendix 2).

The inventory analysis was performed with the LCIA Scores tool (Muñoz-Liesa et al. 2024) using the Ecoinvent v3.10.1 as a background database, while the impact assessment results were obtained using SimaPro 9.6.1. We used ReCiPe as an assessment method with a hierarchical perspective at the midpoint level (Huijbregts et al. 2017). The impact categories considered in this study were as follows: global warming (GW; kg CO_2_ equivalent), terrestrial acidification (TA; kg SO_2_ equivalent), freshwater eutrophication (FE; kg P equivalent), marine eutrophication (ME; kg N equivalent), fossil resource scarcity (FRS; kg oil equivalent), and ecotoxicity (ET; kg 1.4-DB equivalent), which is the sum of terrestrial, freshwater, and marine ecotoxicity.

#### Sensitivity analysis on greenhouse covering material

A sensitivity analysis was carried out to assess the environmental impact of periodically replacing the polycarbonate sheets and thus, improve crop yields over time. Since materials can affect the loss of radiation, increasing it can improve the sustainability assessment. This analysis focused exclusively on the greenhouse covering materials, as these are the only components expected to be replaced in the short term to prevent yield decline. In contrast, the rest of the greenhouse structure (e.g., steel and concrete) has an estimated lifespan of 50 years, as it is often considered for building structures (Ortiz et al. 2009; Zabalza Bribián et al. 2009; Palacios-Munoz et al. 2019), since the i-RTG is an integral part of the building façade (Muñoz Liesa 2022).

To this end, different scenarios were simulated by varying the lifespan of the covering material (from 2 to 6 years: S2–S6, and up to 10 years: S10, as recommended by the product specifications) and adjusting the FU according to projected yields after each substitution.

To assess the impacts of covering material solely, we used the 15-C and 21-G crop cycles as reference models due to the significant drop in transmissivity between them (from 50.9% to 39%). Both cycles had comparable fertilization, irrigation, and plant density (2.69 plants m^−2^), but differed in productivity (20.3 and 10.4 kg m^−2^, respectively). Then, we estimated an annual yield loss of 1.65 kg m^−2^ due to reduced transmissivity, that was used to calculate the average yield for each simulated scenario (Table SM3).

### System experimentation design

In contrast to traditional trials designed to isolate the effect of single variables, this 8-year study was conducted as a system experiment. Following the methodology of iterative design for rule-based systems (Debaeke et al. 2009; Catalogna et al. 2022; Meynard et al. 2023), the objective was to evaluate the feasibility and performance of an evolving management strategy in achieving high tomato productivity while reducing environmental impacts associated to its system. Different crop cycles during the years were managed through a step-by-step design approach, a situated process where “designing by doing” allows for the progressive refinement of the system.

Each cycle functioned as an iterative loop consisting of four activities: diagnosis (e.g., identifying yield gaps due to light loss), exploration (e.g., testing recirculation or struvite), implementation, and assessment. This approach justifies the lack of identical repetition across years, as the management rules were not fixed but adapted based on the learnings from previous cycles. Therefore, the chronological changes in varieties, nutrient concentrations (Table 2), and infrastructure were intentional steps towards optimizing resources and decoupling yield from environmental impact. The LCA methodology was integrated into the assessment phase of the iterative loops to provide a comprehensive evaluation of environmental performances. The inclusion of LCA is common in controlled environment agriculture studies, where changes to the production system to improve sustainability are frequent (Martin et al. 2023).

## Results

### Yield

**Fig. 4:**
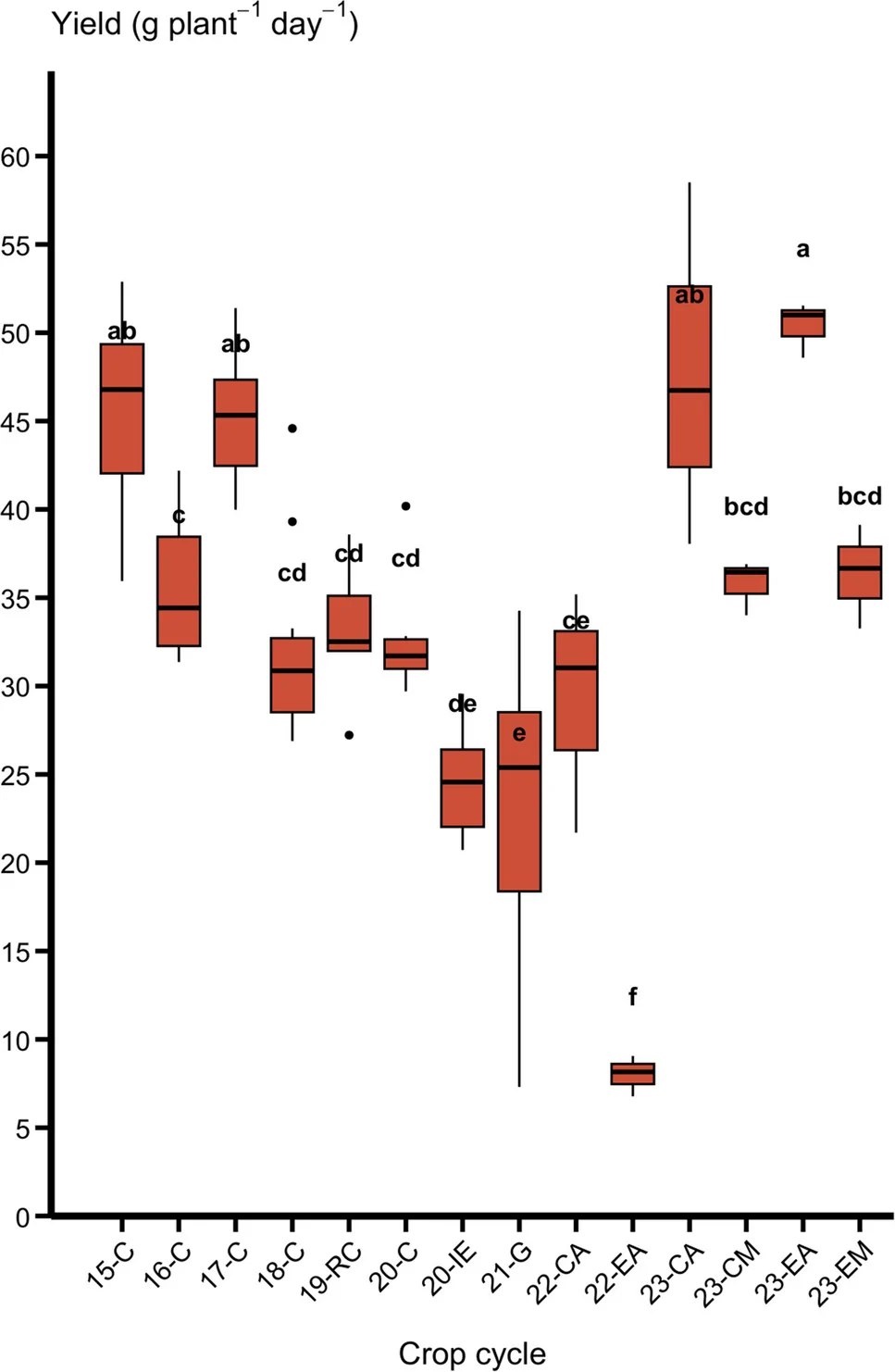
Results of tomato yield expressed in g plant^−1^ day^−1^. Different letters indicate significant differences (*p* < 0.05). C: crop cycle with regular fertilization; RC: recirculation system; IE: indoor environment; G: Gigawak cv.; CA: mineral fertilization and Arawak cv.; EA: struvite fertilization and Arawak cv; CM: mineral fertilization and Montgrí cv.; EA: struvite fertilization and Montgrí cv.

The highest tomato yields (expressed in kg m^−2^) were obtained by 17-C, 15-C, and 20-IE (Table 3), with significant differences found with most of the crop cycles (Appendix B). The lowest yields were obtained by cycles 22-CR, 22-EA, and 22-ER. The highest g plant^−1^ day^−1^ values were reached by 23-CA and 23-EA, with yields of 49 g plant^−1^ day^−1^ for both crop cycles (Fig. 4; Appendix B). These results were probably due to the high LUE obtained (4.37 and 4.30 g mol^−1^, respectively) thanks to the new polycarbonate sheets and the adoption of nutrient solutions that were more suitable for the phenological stage of the plant. The 15-C and 17-C crop cycles resulted in significantly higher yields, with values of 45.5 and 45.2 g plant^−1^ day^−1^, respectively. Again, the yield in these cycles may be explained by high transmissivity and correct fertigation. Statistically, the yields of 15-C, 17-C, 23-CA and 23-EA were significantly higher than those of most crop cycles, except for 23-CM and 23-EM, which showed comparable performance. Statistically lower values occurred in 2022 for the section of the greenhouse fertilized with struvite; values of 10.2 and 3.9 g plant^−1^ day^−1^ were obtained in cycles 22-EA and 22-ER, respectively. These low yields were probably caused by overfertilization with struvite and a lack of potassium and sulphur during flowering and fruiting. The 15-FW crop cycle was also characterized by a significant low yield due to the small amount of solar radiation received during winter (96.8 MJ m^−2^) (Appendix B). However, high LUE values (27.8 g FW mol^−1^) demonstrated that plants were able to utilize lower levels of radiation effectively to achieve a good yield.

There was an overall decrease in yield of 31.2% over 8 years, caused by a reduction in radiation transmissivity due to the deterioration of the polycarbonate sheets covering the i-RTG. Thus, the transmissivity values declined until 2023, when some polycarbonate sheets wrongly installed were replaced due to hail damage, boosting the value up to 1461.5 MJ m^−2^ (46.4% of transmissivity). Notably, the replacement of the polycarbonate sheets — an intentional step toward system optimization — led to a 56.5% increase in yield from cycle 22-CA to cycle 23-CA.

### Water consumption

Analyses of total water consumption and WUE were performed for all the experiments, and the results are displayed in Table 3. Since most of the experiments had different plant densities and days of cultivation, WUE values provide a better understanding of water consumption. The best WUE was achieved in cycle 20-IE; 30.3 g of tomatoes were produced per litre of water consumed. This was due mainly to the stable temperature (21 ± 1.4 °C) and relative humidity (75 ± 10%) of the indoor room, which resulted in lower plant transpiration and reduced consumption of irrigation water. In the i-RTG, the highest WUE was obtained in the 20-IS cycle when water input was reduced, and recirculation was implemented. For this cycle, 4650 L of drained water — representing 31% of total water inputs — were reused for irrigation, raising the WUE from a hypothetical 25.4 (without recirculation) to 36.8 g L^−1^. The poorest WUE was obtained in 2022, when struvite and the Rosa de Cadiz variety were used; this combination resulted in WUE ranging from 2.1 to –7.9 g L^−1^. Low WUE values were related to the low yields and low radiation levels that occurred in the i-RTG during these crop cycles.

### Environmental performance of crop cycles

**Fig. 5:**
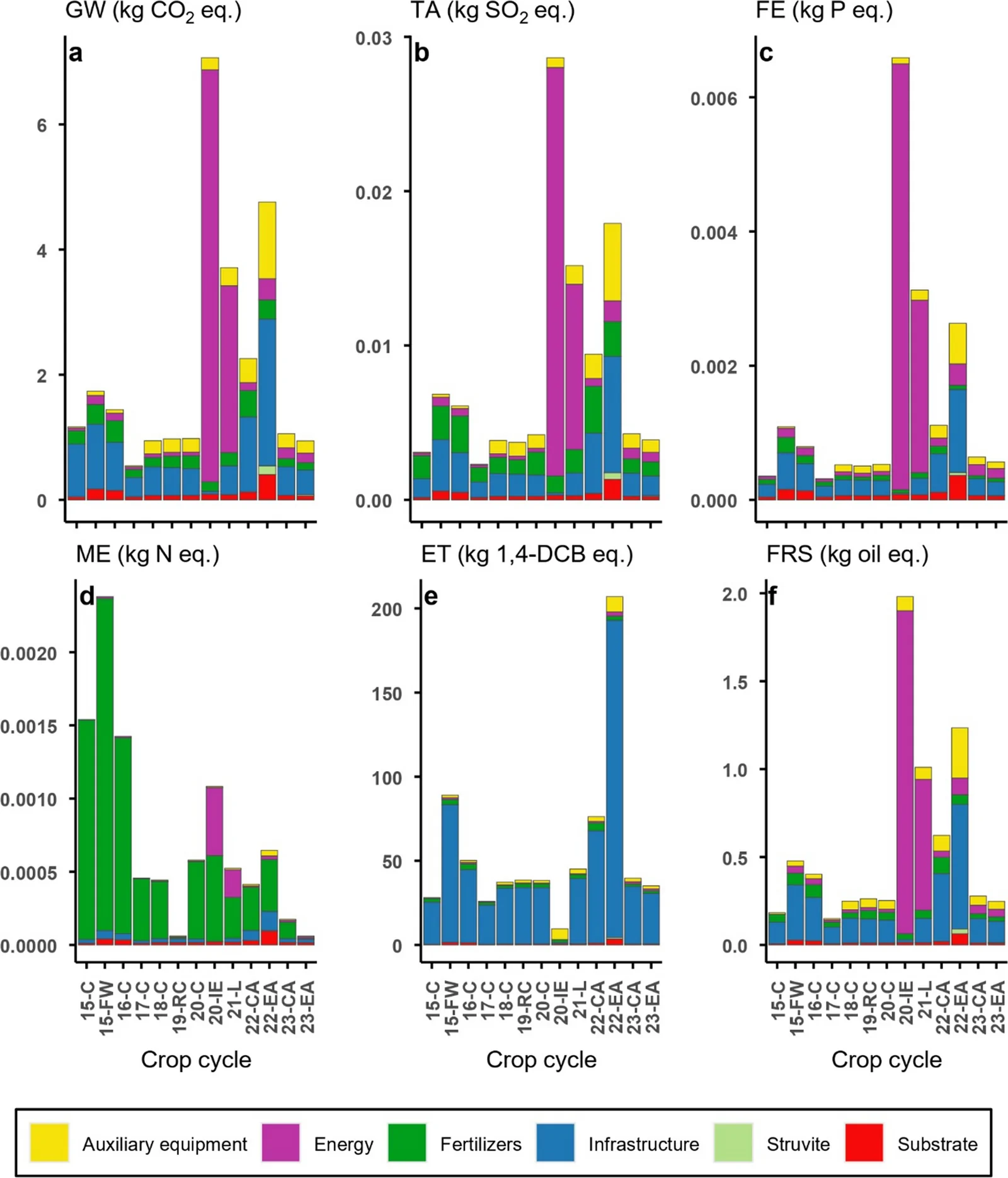
Environmental impact per kg of tomato produced. **a** GW: Global warming; **b** TA: Terrestrial acidification; **c** FE: Freshwater eutrophication; **d** ME: Marine eutrophication; **e** ET: Ecotoxicity; 1,4-DCB: 1,4-Dichlorobenzene; **f** FRS: Fossil resource scarcity; FW: fall-winter cycle; RC: recirculation system; IE: indoor environment; L: supplemental lighting sector; G: Gigawak cv.; CA: mineral fertilization and Arawak cv.; EA: struvite fertilization and Arawak cv. The complete dataset is presented in supplementary information (Figures SM3 and SM4; Appendix 2).

The results presented in this section reveal that radiation, fertiliser, and water management affected yields and environmental impacts. The results were disaggregated into auxiliary equipment (also including tap water processes when needed), fertilizers (also including emissions to the air and water), struvite production, substrate, energy consumption, and infrastructure, which included the rainwater harvesting system and the rooftop greenhouse structure. Figure 5 presents the absolute environmental impact for each category, while the relative contribution of each component to the total impact (expressed in percentage) is available in the supplementary materials (Fig. SM4).

Since the infrastructure remained unchanged, its impact depended on the yield and the duration of a specific crop cycle. Therefore, cycles that produced low yields (expressed in kg m^−2^) showed a high percentage of impact allocated to the infrastructure (e.g., 22-EA). On the other hand, since most inputs from other processes changed for every crop cycle, their environmental impact depended more on fertilization techniques, irrigation management, specific components of the auxiliary equipment, and energy consumption (Table SM2).

In the graph, only the results of the Arawak variety are shown (plus cycles 15-FW and 21-L), since this variety was the most productive and consistent. The Siranzo, Gigawak, and Rosa de Cadiz varieties showed lower yields compared to most crop cycles, since crop management practices were optimized for the Arawak variety and might not have been optimal for the other cultivars. Crop cycles with the Siranzo (21-S) and Gigawak (21-G), varieties characterized by high flower and fruit set, resulted in low yields and consequently high environmental impacts, probably due to non-optimal management practices. On the other hand, the outcomes of the 23-CM and 23-EM cycles were likely influenced by the nature of the Montgrí variety, since these cycles received the same inputs as the 23-CA and 23-EA cycles, which were two of the best cycles in terms of yield and the environmental footprint. Crop cycles with the Rosa de Cadiz cultivar (22-CR and 22-ER) presented low productivity values and consequently high environmental footprints in all categories, partly due to the nature of the variety. However, the low yield obtained in 22-ER (as well as in 22-EA) was probably caused by a lack of potassium and sulphur in the nutrient solution for the last 73 days of the experiment. A complete dataset of the environmental impacts of all crop cycles can be found in the supplementary information (Figures SM3, SM4; Appendix 2).

The experiment that had the lowest impact in all categories, apart from ME, was the 17-C cycle in which the highest productivity (in kg m^−2^) was also obtained. This is despite this experiment did not include any optimization of resources, such as leachate recirculation or struvite fertilization. Thus, a successful outcome was related to a high yield per m^2^ that was obtained with high levels of solar radiation and correct fertigation. On the other hand, the crop cycles with the greatest impacts were 22-EA, 20-IE, and 21-L. While the outcome of 22-EA was influenced by its low yield, the outcomes of 20-IE and 21-L were affected by the energy consumption required for artificial lighting.

High yield and low water and nutrient consumption were the main reasons for the low GW impacts obtained by the 18-C and 20-C cycles. In general, crop cycles grown with closed systems (19-RC, 19-RR, and 20-IS) presented positive results in all impact categories. For TA, FE, and FRS, the impacts followed the same trend as those in the GW category, with the best results obtained by crop cycles with high yields per square metre (15-C, 17-C, 18-C, and 20-C) or recirculation strategies (19-RC, 19-RR, and 20-IS); again, 20-IE, 21-L, and 22-EA accounted for more impacts within these categories. With respect to the ME category, the impacts were driven mainly by fertilizer consumption and consequent emissions to water bodies. For crop cycles that involved recirculation techniques (19-RC, 19-RR, and 20-IS), emissions to water were not considered because of the reutilization or storage of the leachates for other crop cycles, with positive outcomes for the ME category. On average, crop cycles that relied fully on mineral fertilizers in an open system were responsible for an average of 78.8% of the emissions in the ME category. For cycle 15-C, the most impactful crop cycle in this category after 15-FW, the impacts of leachates accounted for the 80.6% of the overall ME impact. To reduce such impacts, the use of 100 g of struvite per plant was effective, as shown by the results of 23-EA: emissions in the leachates were responsible for only 4.5% of the ME impact, making this crop cycle among the most environmentally sustainable for this category.

To compensate the high temperatures of the i-RTG that could affect transpiration and thus, crop yields, a nebulization system was installed in 2023. Even if the 23-CA and 23-EA crop cycles resulted in positive yields, their global warming impacts were negatively affected by energy consumption by the nebulization system. In particular, for 23-CA, the nebulization system was responsible for 34.6% of impacts, mainly due to energy use. Processes related to energy consumption also heavily affected the two crop cycles that employed artificial lighting (20-IE and 21-L). For 20-IE, this process was responsible for 93.1% of the GW impacts, leading this crop cycle to produce 7.06 kg of CO_2_ eq. per kg of tomatoes produced. Similarly, for 21-L, the energy for supplemental lighting accounted for 71.8% of the GW impacts.

The 22-EA crop cycle was among the worst in impact in the GW category. A low yield of 2.3 kg m^−2^ increased the environmental impact to 4.7 kg of CO_2_ eq. per kg of tomatoes produced. This crop cycle was strongly affected by poor light transmission of the polycarbonate sheets, insufficient amounts of potassium, calcium, and sulphur, and an excessive dose of struvite (140 g plant^−1^).

With respect to ET, the environmental impacts were driven primarily by the infrastructure, which was significantly influenced by the low productivity values. Consequently, the best results were obtained by cycles 15-C and 17-C, whereas the crop cycles with the greatest impacts were 22-EA, 15-FW, and 22-CA.

**Fig. 6:**
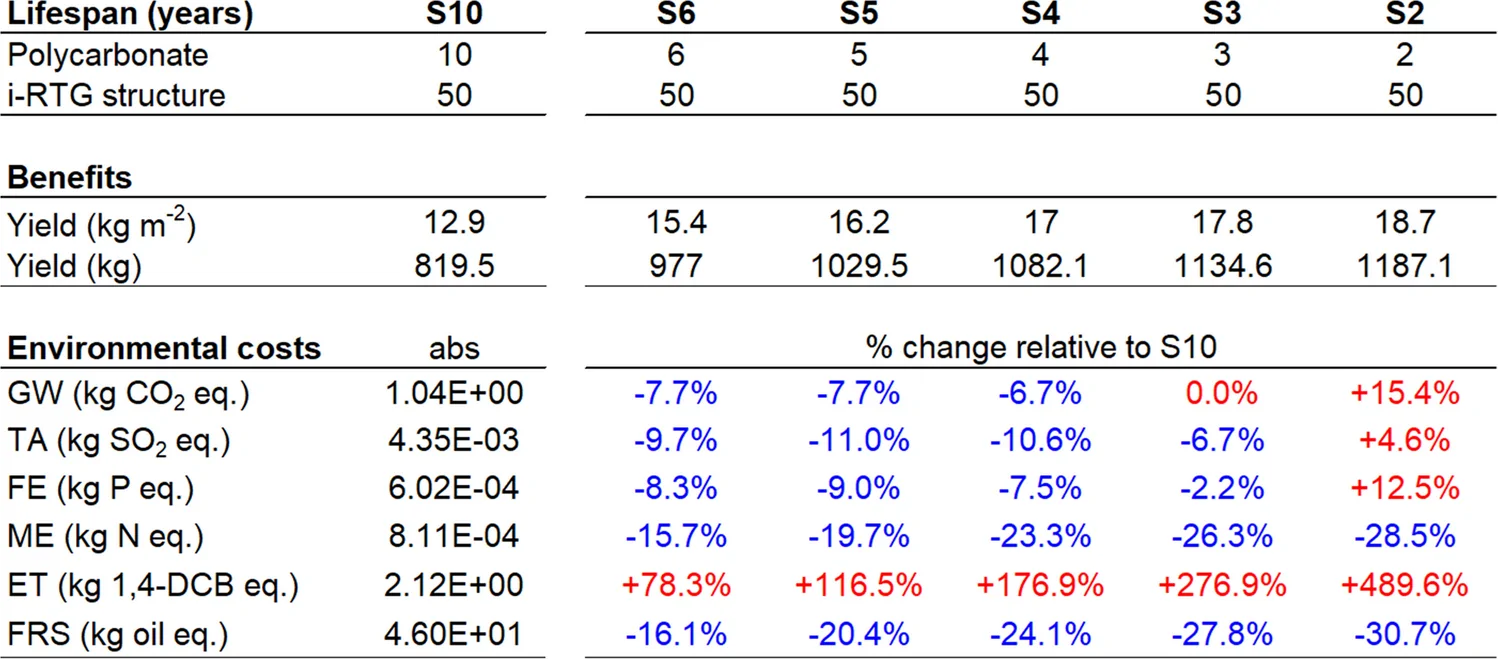
Outcomes of the sensitivity analysis performed on the polycarbonate sheets covering the i-RTG, with negative percentages indicating impact savings. i-RTG: integrated-rooftop greenhouse; GW: global warming; TA: terrestrial acidification; FE freshwater eutrophication; ME: marine eutrophication; ET: ecotoxicity; FRS: fossil resource scarcity; 1,4-DCB: 1,4-Dichlorobenzene.

Using the average yield of various scenarios as the FU, the results of the sensitivity analysis are presented in Fig. 6, while additional data of the environmental impact were included in the supplementary materials (Table SM4).

The results of our analysis show that by substituting the polycarbonate sheets after 4 to 6 years (S6, S5, S4), lower environmental impacts compared to the S10 scenario are found in all impact categories, excluding ET due to the relative contribution of ET impacts from the polycarbonate material (Fig. 6). Oppositely, S10 showed the lowest ET impact, which increased progressively from 78.3 to 489.6% with more frequent replacement of the covering material.

## Discussion

### Solar radiation and nutrient management dominate crop productivity

Based on 8 years of tomato crop cycles, we found that solar radiation and nutrient management were the most influential factors in crop productivity. The highest yields (45.2–45.5 g plant^−1^ day^−1^) were obtained during the summer cycles of 2015 and 2017, when the polycarbonate panels of the greenhouse roof were relatively new and had high transmissivity values of 50.9% and 47.2%, respectively. The partial replacement of polycarbonate panels in 2023 resulted in the highest yields of the entire period (Table 3; Fig. 4), owing to an increase of 6.4% in transmissivity compared with the previous year. The beneficial effect of optimal transmissivity (also reflected in LUE values) on tomato yield is supported by the absence of significant differences between 15-C and 17-C with 23-CA and 23-EA. Together with the CO_2_ concentration in the i-RTG, light has a key impact on plant growth, and its limitation has been shown to negatively affect yield (Montero et al. 2017). This was demonstrated previously in the i-RTG, where a direct correlation between solar radiation and crop yield effects was investigated, resulting into average radiation loss per year of 2.47% (Muñoz-Liesa et al. 2022b). Although the polycarbonate was expected to have a lifespan of 10 years, according to the polycarbonate manufacturing company (Brett 2024), the decay in transmissivity was > 20%. Without enough radiation distributed throughout a crop cycle, growth is slower, and plants produce fewer flowers and fruits. In a study by Kläring and Krumbein (2013), constraining the intensity of solar radiation significantly decreased the photosynthesis, growth, and yield of tomato plants. In conventional greenhouses in the Mediterranean region, the adoption of shading techniques combined with cooling techniques can have positive impacts on the microclimate, thanks to reductions in air temperature by 5–10°C and increases in relative humidity by 15–20%, compared to the outside environment (Ahemd et al. 2016). However, this might not be a good solution in an i-RTG, where the infrastructure and surrounding buildings already limit light transmissivity and interception (Appolloni et al. 2022; Muñoz-Liesa et al. 2022b).

In addition to the transmissivity of the rooftop material, management of the nutrient solution was a key element in maintaining high productivity in the i-RTG. The frequency, dosage, and timing of nutrient supply influenced yield more than the source of the nutrients. Fertilizing tomatoes with nutrients recovered from WWTPs in the form of struvite resulted in yields statistically comparable to conventional fertilization, as shown by experiments 23-EA and 23-EM (Fig. 4). We attributed the high yield per plant in 2023 not only to high radiation but also to new management of the nutrient solution (Table 2). The nutrient solution was adjusted on the basis of the phenological stage of the plant. In fruiting vegetables, the nitrogen concentration should be decreased during flowering and fruiting to increase the fructification process (Savvas et al. 2013). A high nitrogen concentration in nutrient solution can promote the vegetative parts of plants at the expense of the fruit. This could explain the slight differences in yield among the 15-C, 16-C, and 17-C crop cycles, where high levels of nitrates were employed during the fruiting phase (Table 2). A concentration of 7–10 mM L^−1^ of potassium in the nutrient solution is recommended for high fruit production and flavour (Savvas et al. 2008), while low potassium supply (3.5–6 mM L^−1^) during numerous cycles could have led to lower fruit production per plant (Table 2). Generally, the nutrient content of a recirculating solution should be lower than that of an open system, but the concentrations of macronutrients for the 19-RC, 19-RR, and 20-IS cycles were not enough compared with the suggested values for closed systems under Mediterranean climatic conditions (Savvas et al. 2013), leading to reduced productivity.

Another important change made during 2023 was the addition of NH_4_^+^ to the plants. Adding NH_4_^+^ has been shown to decrease the pH in the root zone and produce positive effects on tomato yield (Sonneveld and Voogt 2009; Savvas et al. 2013). However, an excessive amount of ammonium can reduce the uptake of other cations, such as Ca and K and increase susceptibility of the plant to physiological disorders that can undermine fruit quality (Halbert-Howard et al. 2021; Saure 2001). With respect to the 23-EA crop cycle, ammonium provided with 100 g of granulated struvite (~5.7 g of NH_4_^+^) resulted in statistically higher yields relative to the majority of the cycles, while a dose of 140 g (~8 g of NH_4_^+^) administered in the 22-EA cycle may have been a cause of lower fruit set, as shown by the significantly reduced yields produced (Fig. 4). The success of the 2023 cycles suggests that the system reached a peak of optimization through successive refinements of nutrient doses, moving away from the over-fertilization observed in 2022. Our results align with the findings of Carreras-Sempere et al. (2021), who reported a positive correlation between struvite supply through fertigation and tomato fruit yield.

### Environmental benefits depend mostly on yield, fertilization methods and energy consumption

The life cycle impact assessment was highly sensitive to yield, fertilization, and energy use. The lowest impacts across GW, TA, FE, ET, and FRS were obtained in the 17-C crop cycle, in which large quantities of tomatoes were produced, and the fertilization was adjusted based on the needs of the plant. The same cannot be said for the ME category, in which the best outcomes occurred in the 23-EA cycle (with struvite fertilization) and in closed leaching system cycles (19-RC, 19-RR, and 20-IS). For this category, the impact of fertilizers in open system cycles with no recirculation or nutrient recovery methods averaged over 90 times greater than that in experiments with leachate recirculation, in agreement with previous studies performed on tomatoes and green beans (Parada et al. 2021; Rufí-Salís et al. 2020c). However, although leachate recirculation reduced emissions in the ME category, environmental shifts were produced in other categories. In particular, the use of a closed system for cycle 19-RC resulted in greater impacts compared to 17-C, an open-system crop cycle, with increases of 1.79, 1.61, 1.60, 1.48, and 1.75 times observed in the GW, TA, FE, ET, and FRS categories, respectively. This was likely due to the low yield obtained, which was influenced by insufficient nutrients. Recirculating water and nutrients may reduce environmental impacts, but it is crucial to continuously monitor and adjust nutrient levels to ensure that plants receive the optimal amounts needed for each stage of growth. The adoption of struvite as a P source in 2023 strongly reduced impacts in the ME category. In the 23-EA cycle, the impacts were 16.4 lower than those in the 23-CA cycle. These results were obtained because of the small amounts of N and P discharged in the leachates (5.5 and 0.2 g per plant, respectively; Table SM1). Moreover, the P retained in the substrate (38.3%) can be furtherly used for future crop cycles in soilless and open field agriculture (Arcas-Pilz et al. 2022, 2021). The use of this recovered salt was effective, and, owing to the high amount of it found in the substrate at the end of the cycle, lower doses could be used to further reduce the environmental footprint.

Processes related to energy consumption strongly affected the environmental impact of the 20-IE and 21-L crop cycles, which included the use of artificial lights. For FRS, electricity consumption was responsible for 93.1% and 69.9% of the impacts for the 20-IE and 21-L crop cycles, respectively. A comparison of 21-L and 21-S, two crop cycles with the same conditions except for supplemental lighting, revealed that LED lighting increased yield per plant by 41.6% but also increased the GW impact by 132.5%, with similar trends across all impact categories. The assessment phase of the 21-L cycle prompted a shift in optimization objectives toward increasing the radiation via covering material substitution in 2023. With respect to 20-IE, a crop cycle in an indoor environment, even if yield and WUE were among the highest, the use of LEDs strongly influenced the environmental impacts of this crop cycle. High yield may be explained by the stable temperature and humidity during growth. The plants avoided external stresses such as heat peaks and humidity fluctuations, which affect yield or cause plants to produce non-commercial fruits (Sato et al. 2006; Harel et al. 2014; Alsamir et al. 2021). Previous studies have demonstrated that higher yields may be reached in similar settings; for example, for dwarf tomato cultivation via vertical farming, Righini et al. (2023) reported a yield of 36 kg m^−2^ year^−1^. Similarly, Lim et al. (2024) reported that compared with greenhouse cultivation, vertical farming resulted in greater fruit weight and number in dwarf tomatoes. With stable environmental conditions, it is easier to dose the right amount of fertigation, hence avoiding water and nutrient losses through leaching. Our findings align with those of previous studies that demonstrated the positive effects of growing tomatoes indoors on yield and water consumption (Mempel et al. 2021). However, the use of artificial light and climate regulation during indoor tomato cultivation increased the environmental impact across all categories, making it less sustainable than the use of a passive greenhouse. Kobayashi et al. (2022) estimated that producing indoor tomatoes would consume 16–34 kWh per kg produced, which is greater than the amount of energy used in greenhouse cultivation in the Netherlands (0.17 kWh kg^−1^), Spain (3.8–7.6 kWh kg^−1^), Saudi Arabia (8 kWh kg^−1^), and Germany (6.1–12.8 kWh kg^−1^) (Ntinas et al. 2017; Pérez Neira et al. 2018; Torrellas et al. 2012b; Tsafaras et al. 2022). The energy consumption in the 20-IE cycle was 19.7 kWh kg^−1^, which is within the range estimated by Kobayashi et al. (2022) but much greater than the average value obtained in greenhouse experiments without supplemental lighting (0.49 kWh kg^−1^; Appendix 2). Importantly, the energy used to maintain stable environmental conditions in the growing room was excluded from the system boundaries because of a lack of data. Previous studies have shown how this input is a major contributor to all impact categories (Naseer et al. 2022), and it is estimated as an additional 25% of total energy consumption (Kozai 2013a, 2013b). Therefore, by including this input in our dataset, it is likely that this crop cycle would have had the highest environmental impact in all categories. Renewable energy sources could be used to decrease the environmental impact of artificial lights or climate regulation; by changing this input to one that fully relies on photovoltaic energy production, GW impacts could be lowered from 7.06 to 0.93 kg of CO_2_ eq. per kg tomatoes for the 20-IE crop cycle. Moreover, it is important to emphasize that the single 20-IE crop cycle was conducted on only 18 plants in an unusual horticultural setting; hence, this outcome may not be representative and cannot be extrapolated because economies of scale improve system efficiencies.

During 8 years, there were some changes in the auxiliary equipment that hindered our analysis, such as the adoption of aluminium trays and nebulization systems. Replacing plastic trays with aluminium trays for leachate management increased the emissions in all categories due to the high impacts derived from the use of non-renewable energy sources in the aluminium production industry. In crop cycles 16-C and 23-CA, which had similar productivity values (13.9 and 12.9 kg m^−2^, respectively), the aluminium trays had an impact that was 54.4 times greater for the ET category than the plastic trays did. Similar trends occurred in other crop cycles following the introduction of aluminium trays in 2018. With respect to the nebulization system, the energy input negatively influenced the results of crop cycles in 2023. Our data showed that for 23-CM and 20-C (two cycles with similar kg m^−2^ and processes), the addition of the nebulization system increased GW emissions by 69.3%. This addition was necessary to decrease the temperatures and increase the humidity during episodes of extreme heat to mitigate the adverse effects that supra-optimal temperatures can have on pollination, blossoming, fruit set, and quality (Heusden and Lindhout 2018; Heuvelink and Okello 2018; Kubota et al. 2018). To partly solve this problem, renewable energy mixes could be adopted to decrease emissions in most categories.

By comparing our results with past studies on conventional greenhouses in the Mediterranean, the impact of tomato produced in an i-RTG is often higher. In a study conducted by Cellura et al. (2012) with a cradle-to-grave approach, tomato production in a pavilion greenhouse in Italy resulted in emissions of 0.74 kg CO_2_ eq. per kilogram of produce. In multi-tunnel greenhouses, cherry tomato production in Tunisia generated 0.95 kg CO_2_ eq. (Maaoui et al. 2021), while truss tomato cultivated in Almeria (Spain) only produced 0.25 kg CO_2_ eq. (Torrellas et al. 2012a), with both studies adopting a cradle-to-gate perspective. The lowest emissions in the i-RTG were recorded by 17-C (0.54 kg CO_2_ eq.), with the infrastructure alone accounting for 56.5% of the total emissions in the GW category. This value suggests that with a conventional greenhouse structure, the overall impact of our system could likely have been lower than those reported in the cited studies.

Plant density also influenced the impact outcomes. Beginning in 2022, the experiments were designed with a density of two plants per bag (1.79 plants m^−2^), in contrast to previous years (2.69 plants m^−2^). By occupying the same space with fewer plants, the impacts related to the greenhouse structure and the auxiliary equipment (such as the aluminium trays) increased because the allocation factor based on the occupied area. While it is common practice to adopt a density of 2.5 plants m^−2^ (Heuvelink et al. 2018), higher densities could be reached to increase yield (Maboko et al. 2011). Moreover, increasing plant density could have positive effects on the microclimate by increasing the relative humidity and reducing the temperature, which could decrease the need for a nebulization system. However, a higher plant density reduces light interception due to reciprocal shading, which, in addition to the limited transmissivity in the i-RTG, might negatively affect yield in the long term.

### Changing polycarbonate sheets from 4 to 6 years brings both yield and environmental benefits

Results of the sensitivity analysis suggest that, to maintain high yields and low environmental impacts, polycarbonate sheets should be changed within 4 to 6 years (Fig. 6), instead of 10 years as suggested by the manufacturing company (Brett 2024). This is probably worsened by the fact the initial i-RTG solar transmissivity was already low due to the building shadows (50.9%), while higher values should be obtained by using the same material in conventional settings (83%) (Brett 2024). Moreover, in conventional greenhouses in warm climates, polyethylene films — the most popular covering materials — are usually substituted every 2 to 4 years to ensure optimal radiation since the transmissivity decaying rates are also high (Montero et al. 2013; Reddy 2016; Teitel et al. 2018). Although the materials and lifespans differ, this highlights a broader principle: as in conventional greenhouses, rooftop greenhouses also benefit from the regular replacement of covering materials to sustain light transmissivity and crop performance. Our simulated scenarios did not include any nutrient or water recovery techniques — such as struvite fertilization — suggesting that, by adding these strategies, the overall environmental impact could be lowered without compromising the productivity.

### Learning loops and transition pathways

The evolution of management strategies over eight years reflects a step-by-step change pathway consistent with iterative system design and adaptive management (Meynard et al. 2023). What could be interpreted as a lack of experimental repetition instead corresponds to successive learning loops applied to the management of a complex system.

For example, the low yields observed during the 2022 crop cycles (22-EA, 22-ER), associated with over-fertilisation with struvite, provided a diagnostic insight that generated a double-loop learning process. This led to a revision of struvite doses in 2023, resulting in the highest productivity recorded in the dataset (49 g plant^−1^ day^−1^). Similarly, the high impacts associated with energy consumption from LED lightings (21-L) prompted a shift in optimisation objectives, from increasing LUE through supplemental lighting toward the substitution of greenhouse covering materials in 2023.

This rule-based and adaptive approach enabled a transition from rigid technology adoption toward flexible management strategies that integrate scientific objectives with real greenhouse constraints. The eight-year trajectory illustrates that, in this context, reliable knowledge is generated not solely through static repetition of identical treatments, but through the systematic testing and refinement of management rules over time. This perspective supports the view that sustainable outcomes in controlled-environment agriculture emerge from successive improvements in resource optimisation rather than from isolated, one-time interventions.

### System limitations

The results presented in this article offer a wide range of information on tomato cultivation in a rooftop greenhouse. Considering the variables that are involved, standardization of cultivation is very complicated, especially in different climatic zones. For example, case studies conducted at higher latitudes might need additional inputs — such as heating in colder months — that can significantly influence the environmental performance of crop cycles. Additionally, the limited number of samples, repetitions (and sometime space) for some studies (e.g. 20-IE, 21-G) as well as reduced technical staff due to COVID-19 restrictions might have negatively influenced the outcomes of some crop cycles. We discussed the influence of the main variables, but other key features may have influenced the crop cycles, such as the work force, management of pruning and pollination. These variables were not evaluated and may have affected the outcomes of the crop cycles.

## Conclusions

By analysing eight years of cultivation through an iterative design framework, this study allows us to draw some conclusions on sustainable tomato cultivation in an integrated-rooftop greenhouse. Our results show that the environmental assessment were sensitive to the functional unit, solar transmissivity, nutrient solution, irrigation technique, and general management of the crop.

The use of 100 g of struvite per plant can reduce the environmental impact related to mineral fertilizers and decrease phosphate discharge to water bodies, while also producing the highest yields for both the Arawak and the Montgrí varieties (49 and 36.6 g m^−2^ day^−1^, respectively) — as confirmed by our learning loops which corrected previous overfertilization. Our findings demonstrates that sustainable outcomes in similar settings are achieved not through isolated interventions, but through the systematic testing and refinement of management rules over time.

The adoption of recirculation strategies will reduce water consumption, decrease the amount of leachate, and prevent the contamination of water bodies; however, attention should be given to providing optimal nutrients to the plants and avoiding salt accumulation in the nutrient solution, which has negative consequences for yield and quality. The use of the recirculation solution or the purged water for cascade crops should also be considered, with the possibility of allocating the environmental impact among multiple crop cycles.

Replacing the polycarbonate sheets every 4 to 6 years, to ensure optimal solar transmissivity, results in higher productivity (19.4–31.8% increase) and lower environmental impacts (6.7–7.7% reduction in GW impact) compared to the 10-year replacement interval recommended by the manufacturer.

It is unclear whether cultivation in an indoor environment is environmentally unsustainable based on only one cycle with limited plants. Better environmental performance requires the implementation of clean energy in lighting and other processes (e.g., ventilation). Additionally, further studies should investigate the optimal tomato variety and management for indoor settings.

Future studies should focus on testing our findings in different climatic zones and locations, as well as applying alternative nitrogen sources to further reduce the emissions related to mineral fertilizers application. This study provides a wide range of results and alternatives for tomato growth in an integrated-rooftop greenhouse in the Mediterranean region, and our findings can be used to develop sustainable and circular solutions for crop cultivation in similar settings.

## Supplementary Information

Below is the link to the electronic supplementary material.

Supplementary Material 1 (DOCX 1.07 MB)

Supplementary Material 2 (XLSX 134 KB)

Supplementary Material 3 (PDF 79.3 KB)

## Data Availability

Additional data not present in the supplementary information can be requested to the corresponding author.
