# Supplementary material for: Sustainable resource optimization for tomato cultivation in a rooftop greenhouse: an 8-year case study
Source: Agron Sustain Dev. 2026 Jun 23;46(4):49. doi: 10.1007/s13593-026-01118-6 (PMC13291085; doi:10.1007/s13593-026-01118-6)
Supplement: Supplementary file 1 — Supplementary Material 1 (DOCX 1.07 MB) [file 13593_2026_1118_MOESM1_ESM.docx]

Supplementary information

**Appendix 1A. Pictures of the growing structures for the integrated rooftop greenhouse and the indoor environment.**


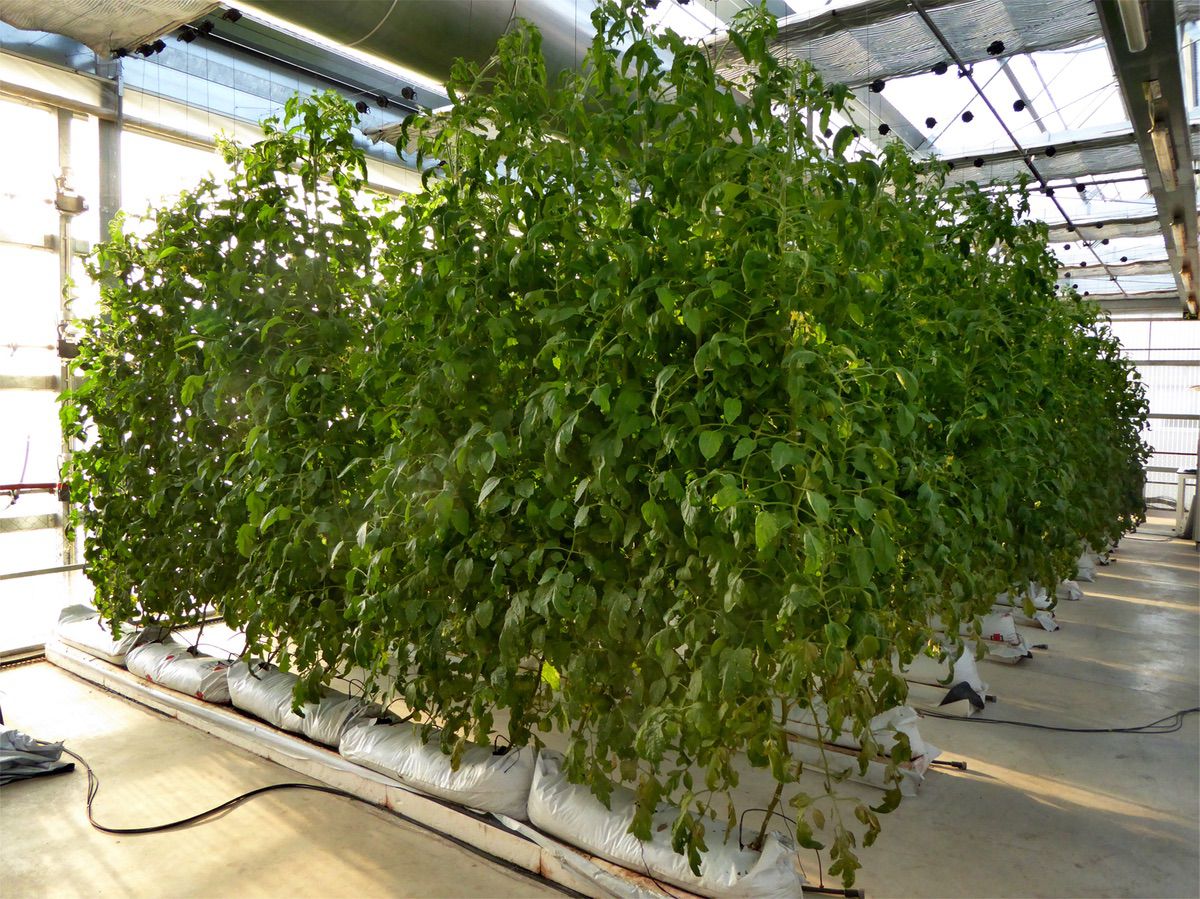

Fig. SM1. Tomato cultivation in the i-RTG for 15-C, 15-FW, 16-C and 17-C crop cycles, characterized by a density of 3 plants per substrate bags and plastic trays for leachate disposal.


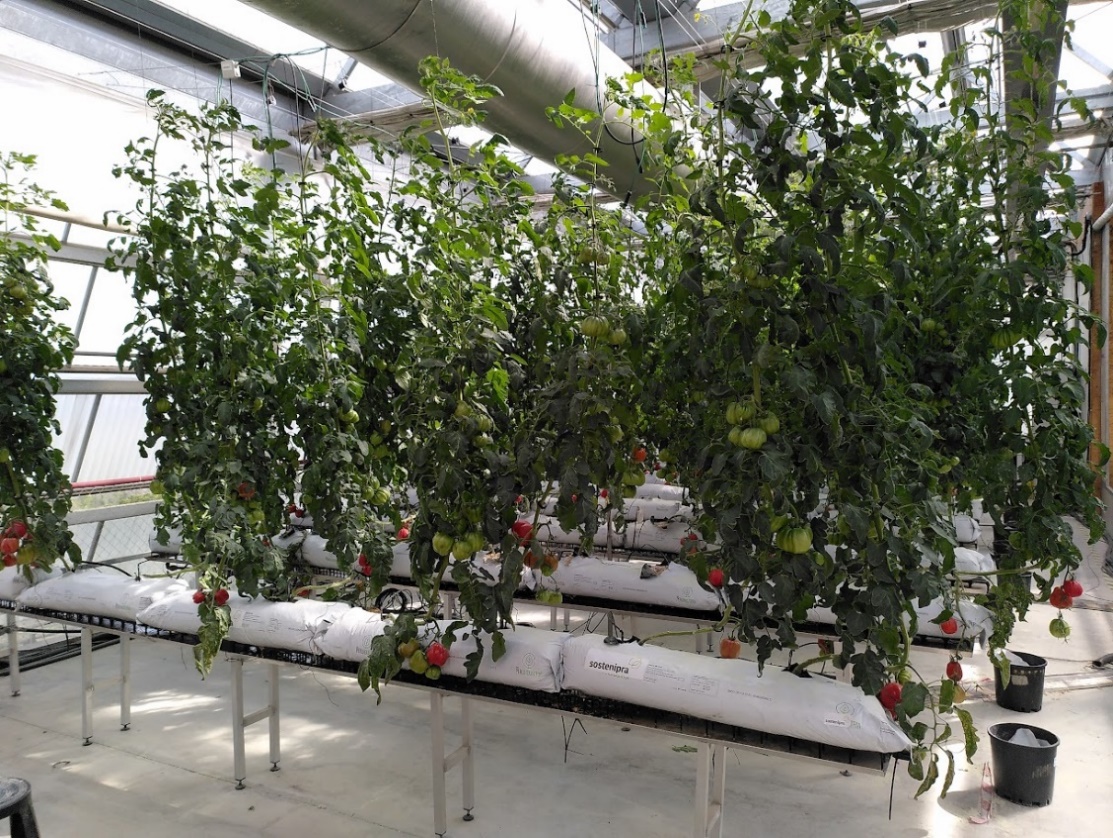


Tomato cultivation in the i-RTG from 2018 on, characterized by the adoption of aluminium trays for leachate management (2023 crop cycle in the picture, with a density of 2 plants per substrate bag).


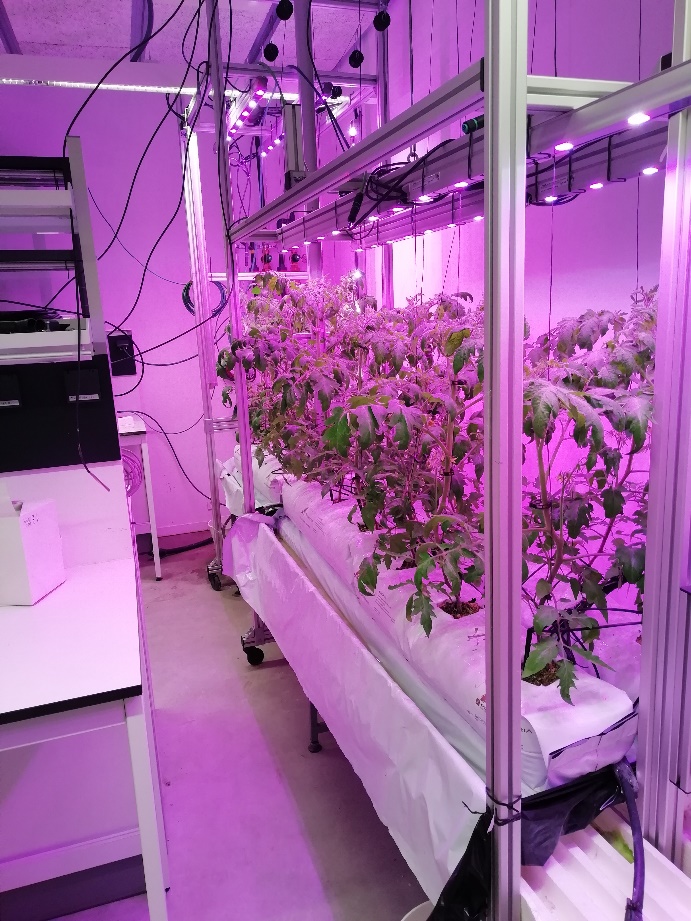

Fig. SM2. Tomato cultivation for the 20-IE crop cycle located in the basement of the ICTA-UAB building and characterized by the adoption of artificial lighting.

**Appendix 1B. Extended methodology of all crop cycles analysed in this study.**

Experiments 15-C, 15-FW, 16-C. The aim of the three experiments set between 2015 and 2016 was to evaluate the environmental assessment of tomato production in an i-RTG and compare the results with conventional greenhouse production. Three crop cycle were performed from February 2015 to July 2016, with one experiment set in the between September and March (15-FW) to understand the feasibility of the cycle during the fall-winter months. For all the experiments, 171 plants were cultivated within 12 rows and with a density of 3 plants per bag of perlite. Additional information about the experiments can be found in the published articles (Sanjuan-Delmás et al., 2018, 2019; Rufí-Salís et al., 2020).
Experiment 17-C. For this experiment, 171 tomato plants distributed among 12 rows were grown from January to July, with a density of 3 plants per perlite bag. Differently from other studies, six distinct nutrient solution mixes were employed. More comprehensive information can be read in the published paper (Rufí-Salís et al., 2020).
Experiments 18-C, 19-RC, 19-RR. The aim of these experiments was to adopt water saving practices for irrigation by recirculating the leachates, while maintaining productivity and reducing environmental impacts. During 2018, 171 plants were grown in an open hydroponic system without strategies to recover the leachates (18-C). The following year, the greenhouse was divided in two areas: 1) for the first half (90 plants), 30% of the drained water was used to irrigate the crop (19-RC); 2) the rest of the greenhouse (81 plants) had the same recirculation scheme but with a further reduction in water input of 15% (19-RR). The content of the nutrient solution was the same among all the experiments and the plant density was of 3 plants per substrate bag. Additional information can be consulted in the published paper (Parada et al., 2021).
Experiment 20-C, 20-IS. The focus of this research was to reduce water consumption with the adoption of an irrigation scheme. To achieve this, the greenhouse was divided into two areas with varying water volumes: 90 plants received regular irrigation (20-C), while 81 plants followed a reduced irrigation schedule (20-IS). The same nutrient solution used for the previous two experiment was adopted and the density was of 3 plants per perlite bag. Due to the breaking of COVID-19 pandemic in March, there were challenges in maintaining consistent data records and regularly managing the greenhouse.
Experiment 20-IE. The aim of this experiment was to determine the feasibility in terms of production, water and energy consumption of a tomato cycle in an indoor environment and compare the results with the test set in the i-RTG during the same months. It was performed using two rows of crops in an indoor room (for total of 18 plants in 6 bags of perlite) with an effective harvesting area of 3.33 m^2^, located in the basement of the ICTA-UAB building and equipped with sensors monitoring temperature (21 ± 1.4 °C), humidity (75 ± 10%) and CO_2_ concentration (500 ± 50 ppm). The crops were fertigated four times a day for 5 minutes during the first 10 days and, for the rest of the cycle, the irrigation scheme was switched to four times a day for 7 minutes. The composition of the nutrient solution was the same of the experiment performed in the rooftop greenhouse. Plants were irradiated using Green Power LEDs Philips system with varying light regimes: the first light mix consisted in 20% Blue, 8% Green, 75% Red and 15% Far-red, with an average intensity of 270 µmol m^-2^ s^-1^ and a daily light integral of 14 hours; after 10 days, the light was switched to 10% blue, 8% green, 80% red, and 20% far-red with an intensity of 220 µmol m^-2^ s^-1^ and the DLI was increased to 16 hours a day; after 20 days of exposure, the radiation was increased to an average of 350 µmol m^-2^ s^-1^ by adding six more LEDs modules. Similarly to the experiment set in the rooftop greenhouse, data collection and plant administration were affected by the restrictions caused by the pandemic.
Experiments 21-L, 21-S, 21-G. The purpose of this experiment was to identify the benefits and limitations of supplementary LED lighting on the yield and quality of tomatoes (cv. “Siranzo”). Sixty plants of tomato (cv “Siranzo”) were distributed in four rows while, in the remaining rows, buffer plants of Siranzo (30 plants) and Gigawak (81 plants) were planted to avoid light treatment pollution among the blocks. Three LED lighting treatments were applied in addition to natural sunlight: red and blue, red and blue + far-red throughout the day, and red and blue + far-red, with far-red applied at the end of the day for 30 minutes. Each light treatment lasted for 16 hours per day with an intensity of 170 μmol m^−2^ s^−1^. Similarly to previous campaigns, the chosen density was of 3 plants per bag of perlite. Additional information on this experiment can be found in the previously published paper (Appolloni et al., 2022).
Experiments 22-CA, 22-CR, 22-EA, 22-ER. The main objective of the experiment was to apply struvite (NH_4_MgPO_4_·6H_2_O) to part of the crops and compare the outcomes with plants grown using mineral fertilizers. Apart from the “Arawak”, “Rosa de Cadiz” variety was also tested. The greenhouse was divided in two sections: 1) a sector consisting of five rows (30 plants of “Arawak” and 20 plants of “Rosa de Cadiz”), receiving 100% mineral fertilizer with drip irrigation (22-CA, 22-CR), and 2) another five rows of plants (26 plants of “Arawak” and 18 plants of “Rosa de Cadiz”) that received a nutrient solution deficient in phosphorous and magnesium and with less N inputs, which were supplied to the crops through 140 g of struvite grains per plant directly placed in the substrate (22-EA, 22-CR). The macronutrient composition varied between the two sectors, as shown in table 2. Specifically, the nutrient solution applied to plants fertilized with struvite had lower levels of nitrates, potassium, calcium, and sulphate compared to the other sector. The struvite was applied three times during the crop cycle, firstly with an initial dose of 20 g (18^th^ of March) and then with two doses of 60 g (13^th^ of May and 10^th^ of June).
Due to a technical issue, from the 12^th^ of May to the end of the cycle, SO_4_ and K were not provided to the plants fertilized with struvite. Compared to previous years, the number of plants per bag of perlite was reduced from three to two, for a total of 94 plants.
Experiments 23-CA, 23-CM, 23-EA, 23-EM. The objective of this research was to compare the yield, quality and environmental assessment of two tomato varieties grown with struvite, with plants fertilized with mineral fertilizers. During the experiment, part of the polycarbonate sheets covering the greenhouse were replaced after being damaged by hail. The chosen varieties were “Arawak” and “Montgrí”. For this experiment, 56 plants received a complete mineral solution (23-CA, 23-CM) while the other 58 received a nutrient solution lacking P, Mg^2+^ and NH_4_^+^, supplied through struvite grains (23-EA, 23-EM). With the knowledge gained during the previous experiment, the dose of struvite was reduced to 100 g per plant applied in two stages: at transplant (60 g) and on April 13^th^ (40 g). Three distinct nutrient solution mixes were employed for both fertilization treatments, with a different dose of micronutrients compared to previous campaigns. Differently from 2022, the values of the main macronutrients (NO_3_^-^, K^+^ and Ca^2+^) in the nutrient solution were almost equal between the treatments. Similarly to the previous year, for this experiment the chosen density was of two plants per substrate bag, for a total of 114.

**Appendix 1C. Methodology for samples analysis to assess the nutrient content in water, plant organs and perlite.**

Samples of irrigation water and leachates were analysed with ion chromatography (ICS-1000 and AS-DV by Dionex) and Eq. (SM1) was used to estimate the total amount of nutrients during the crop cycle.

Total amount of nutrients in the irrigation or leachates during the crop cycle [kg]
$=\frac{\sum\left[ X_{i}*\left( Nc \right)_{i} \right]}{{10}^{6}}$ (Eq. SM1)

Where *i* represent the specific period of time, X_i_ the amount of partial volume (L) in the period *i* and Nc_i_ is the nutrient concentration expressed in mg × L^-1^. In this way, the total amount of nutrients in the irrigation water or leachates, can be obtained by the sum of the multiplication of the partial concentration by the volume, as seen in Parada et al., (Parada et al., 2021).
To assess the content of P and K in samples of biomass, fruits, perlite and roots, analyses were carried out through an acid digestion process with HNO_3_ and analysed using ICP-OES optical spectrometry (Optima 4300DV, Perkin-Elmer, Waltham, MA, USA). The total amount of K and P was calculated using Eq. (5), where DM_i_ represents the partial dry matter of the analysed sample [g _dry matter_], and Nc_DM_ _i_ is the nutrient concentration of the sample [mg∙g^-1^ _dry matter_].

Total amount of potassium and phosphorous in biomass, fruits, perlite and roots [kg]
$=\frac{\sum{DM}_{i}* \left[ {Nc}_{DM} \right]_{i}}{{10}^{3}}$ (Eq. SM2)
Content of nitrogen was evaluated by analysing dry samples of biomass with CHNS analysis (Flash EA 2000 CHNS, Thermo Fisher Scientific) and its amount was calculated using Eq. (6) where DM_i_ represents the partial dry matter of the sample [g _dry matter_] and %N_DM_ represents the percentage of nitrogen present in the dry sample.

Total amount of nitrogen in biomass, fruits, perlite and roots [kg]
$= \frac{\sum{DM}_{i}* \left[ {\%N}_{DM} \right]_{i}}{{10}^{3}}$ (Eq. SM3)

Analysis on perlite samples were performed for 15-C, 15-FW, 16-C and 2023 crop cycles while, for other studies, the values derived based on the percentages obtained during the 2023 experiment. Nitrogen emissions to air (NH_3_, N_2_O and NO_X_) were calculated according to the IPCC standards (IPCC, 2019).

**Appendix 1D. Nutrient balance**

Nutrient balance calculations were performed on N, P, and K for most experiments using equations SM1, SM2 and SM3 and the results are presented in Table SM1.
Nitrogen content in the leachates varied between 8.5% and 50.9%, with the highest value obtained in the 15-C crop cycle, when also the N inputs were the highest (10.5 Kg of total N). The lowest values of nitrogen in the leachates were found in 23-CA and 23-CM cycles, while a big portion of nitrogen were allocated to the biomass (53.4%) and fruits (9.7%). Regarding the leachates, levels of phosphorous ranged between 12.7% and 31.4%, with the largest per-centage obtained again during 15-C. These values did not include the crop cycles with struvite fertilization that, for the 2022 and 2023 campaigns, resulted in low values of phosphates especially in the leachates, due to the slow-release nature of the fertiliser. In the 23-EA campaign, P values of leachates in the struvite sector were 89.4% lower compared to the sector with mineral fertilization while, for fruits and biomass, the P contents were 8.3% and 31.1% smaller, respectively. Phosphorus levels in the fruits remained relatively constant, with values falling within the range of 8.4% to 18.8% while, for the biomass, values ranged from 24.4% to 60.9%. Nitrogen content in tomato fruits varied throughout the campaigns, ranging between 7.6% and 31% while, for the biomass, levels of N fluctuated between 14.9% and 56.4%. Potassium levels in the fruits varied between 5.5% to 22.1%, whereas for the biomass values ranged from 8.9% to 39.8%. The presence of a high content of potassium in tomato fruits is crucial for product quality, particularly in enhancing attributes like size, flavour, firmness, and colour. The percentage of K loss through the leachates remained high through all the experiments, with values that varied between 21.8% and 53.9%.
Values of emissions to the air (X_E_) can be found in the life cycle inventory in the Appendix 2.

Table SM1. Nitrogen, phosphorous and potassium inputs and outputs.
* All the phosphorous and part of the nitrogen (as NH_4_^+^) was applied as struvite grains in the substrate.

| Experiment | Nutrients | Input (g plant^-1^) | Output (g plant^-1^) | | | | | Output g Kg^-1^ DW | |
| --- | --- | --- | --- | --- | --- | --- | --- | --- | --- |
|  |  | Fertilization *(X_T_)* | Leachates *(X_L_)* | Biomass *(X_B_)* | Fruits *(X_F_)* | Perlite *(X_P_)* | Roots *(X_R_)* | Fruits | Biomass |
| 15-C | N | 61.7 | 31.4 | 9.2 | 10.2 | 3.8 | 0.1 | 23.0 | 27.0 |
|  | P | 13.2 | 4.2 | 4.2 | 2.5 | 0.8 | 0.9 | 6.0 | 12.0 |
|  | K | 141.9 | 76.5 | 16.5 | 18.1 | 0.0 | 8.4 | 40.0 | 54.0 |
| 15-FW | N | 16.5 | 6.4 | 7.8 | 3.0 | 1.1 | 0.0 | 24.0 | 39.0 |
|  | P | 6.0 | 1.5 | 3.7 | 0.8 | 0.4 | 0.4 | 6.0 | 18.0 |
|  | K | 52.1 | 23.7 | 13.7 | 5.9 | 0.0 | 3.1 | 47.0 | 69.0 |
| 16-C | N | 21.6 | 9.7 | 8.8 | 6.7 | 1.2 | 0.1 | 29.0 | 32.0 |
|  | P | 7.6 | 1.9 | 2.9 | 1.3 | 0.7 | 0.5 | 4.0 | 11.0 |
|  | K | 53.2 | 28.4 | 13.9 | 10.8 | 0.6 | 3.1 | 33.0 | 59.0 |
| 18-C | N | 37.3 | 8.1 | 13.7 | 9.1 | 0.1 | 0.5 | 26.3 | 22.7 |
|  | P | 13.7 | 2.5 | 5.0 | 2.1 | 1.0 | 0.7 | 6.0 | 7.2 |
|  | K | 97.8 | 38.4 | 29.0 | 17.1 | 5.8 | 1.8 | 48.8 | 50.8 |
| 19-RC | N | 27.8 | 4.6 | 10.6 | 6.1 | 0.1 | 0.4 | 26.2 | 22.5 |
|  | P | 14.1 | 3.6 | 3.4 | 1.4 | 1.0 | 0.2 | 6.0 | 7.3 |
|  | K | 63.7 | 19.1 | 23.0 | 12.7 | 3.8 | 1.2 | 54.2 | 49.1 |
| 19-RR | N | 25.7 | 4.3 | 10.7 | 6.2 | 0.1 | 0.3 | 25.7 | 23.7 |
|  | P | 13.1 | 3.3 | 3.5 | 1.4 | 0.9 | 0.7 | 5.5 | 7.0 |
|  | K | 58.6 | 17.9 | 23.3 | 13.0 | 3.5 | 1.1 | 52.4 | 51.3 |
| 21-L, 21-S,  21-G | N | 16.3 | 3.8 | 6.7 | 3.5 | 0.0 | 0.2 | 24.6 | 31.8 |
|  | P | 6.1 | 0.8 | 2.03 | 0.7 | 0.4 | 0.3 | 4.2 | 4.6 |
|  | K | 55.3 | 20.2 | 17.5 | 3.63 | 3.3 | 1.0 | 45.8 | 44.3 |
| 22-CA, 22-CR | N | 14.1 | 3.9 | 8.0 | 3.2 | 0.0 | 0.2 | 24.7 | 33.2 |
|  | P | 4.8 | 1.3 | 2.2 | 0.7 | 0.3 | 0.2 | 5.2 | 7.9 |
|  | K | 102.5 | 36.2 | 10.5 | 5.6 | 6.1 | 1.9 | 42.7 | 41.1 |
| 22-EA, 22-ER * | N | 18.5 | 6.6 | 4.6 | 1.4 | 1.1 | 0.3 | 28.4 | 32.9 |
|  | P | 16.1 | 0.2 | 1.0 | 0.3 | 6.2 | 0.3 | 5.7 | 7.5 |
|  | K | 6.7 | 1.5 | 0.6 | 1.8 | 0.5 | 0.1 | 35.2 | 5.1 |
| 23-CA, 23-CM | N | 38.3 | 3.3 | 20.5 | 3.7 | 0.1 | 0.5 | 34.6 | 28.8 |
|  | P | 8.4 | 1.9 | 4.5 | 1.2 | 0.6 | 0.4 | 8.8 | 7.2 |
|  | K | 92.2 | 20.2 | 24.3 | 11.3 | 5.5 | 1.7 | 79.6 | 36.7 |
| 23-EA, 23-EM * | N | 42.2 | 5.5 | 16.8 | 5.0 | 2.5 | 0.6 | 35.3 | 29.2 |
|  | P | 12.6 | 0.2 | 3.1 | 1.1 | 4.8 | 0.3 | 7.4 | 6.3 |
|  | K | 87.0 | 24.6 | 18.0 | 11.5 | 7.1 | 1.4 | 80.2 | 34.9 |

**Appendix 1E. Additional information and results of the life cycle impact assessment**

Table SM2. Main differences of the inventory among the experiments.

| Experiment | Plastic  trays | Aluminium trays | LED light | Recirculation  system | Ventilation  system |  |
| --- | --- | --- | --- | --- | --- | --- |
|  |  |  |  |  |  |  |
| 15-C | • |  |  |  |  |  |
| 15-FW | • |  |  |  |  |  |
| 16-C | • |  |  |  |  |  |
| 17-C | • |  |  |  |  |  |
| 18-C |  | • |  |  |  |  |
| 19-RC, 19-RR |  | • |  | • |  |  |
| 20-C |  | • |  |  |  |  |
| 20-C, 20-IS |  | • |  | • |  |  |
| 20-IE |  | • | • |  |  |  |
| 21-L |  | • | • |  |  |  |
| 21-S, 21-G |  | • |  |  |  |  |
| 22-CA, 22-CR, 22-EA, 22-ER |  | • |  |  |  |  |
| 23-CA, 23-CM, 23-EA, 23-EM |  | • |  |  | • |  |

In Fig.SM3 and Fig.SM4 are presented the results of the life cycle impact assessment of all crop cycles, with emission values and their contribution.


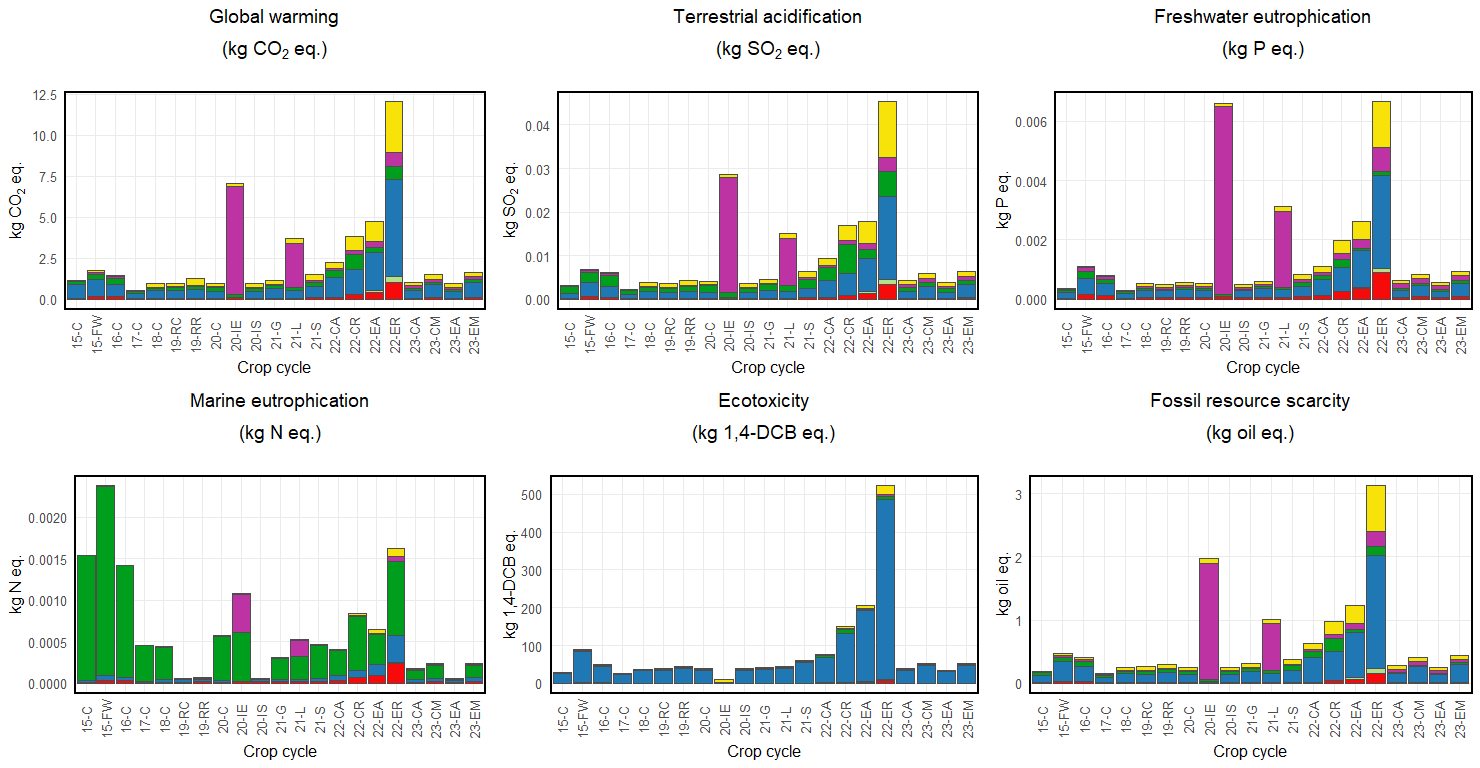

Fig. SM3. Environmental impact per kg of tomato produced of different processes. FW: fall-winter cycle; RC: recirculation system; RR: recirculation system and irrigation reduction; IM: irrigation module; IE: indoor environment; L: supplemental lighting sector; G: Gigawak cv.; CA: mineral fertilization and Arawak cv.; CR: mineral fertilization and Rosa de Cadiz cv.; ER: struvite fertilization and Rosa de Cadiz cv.; CM: mineral fertilization and Montgrí cv; EA: struvite fertilization and Arawak cv; EM: struvite fertilization and Montgrí cv. The exact amount of the impacts can be consulted in the Appendix 2.


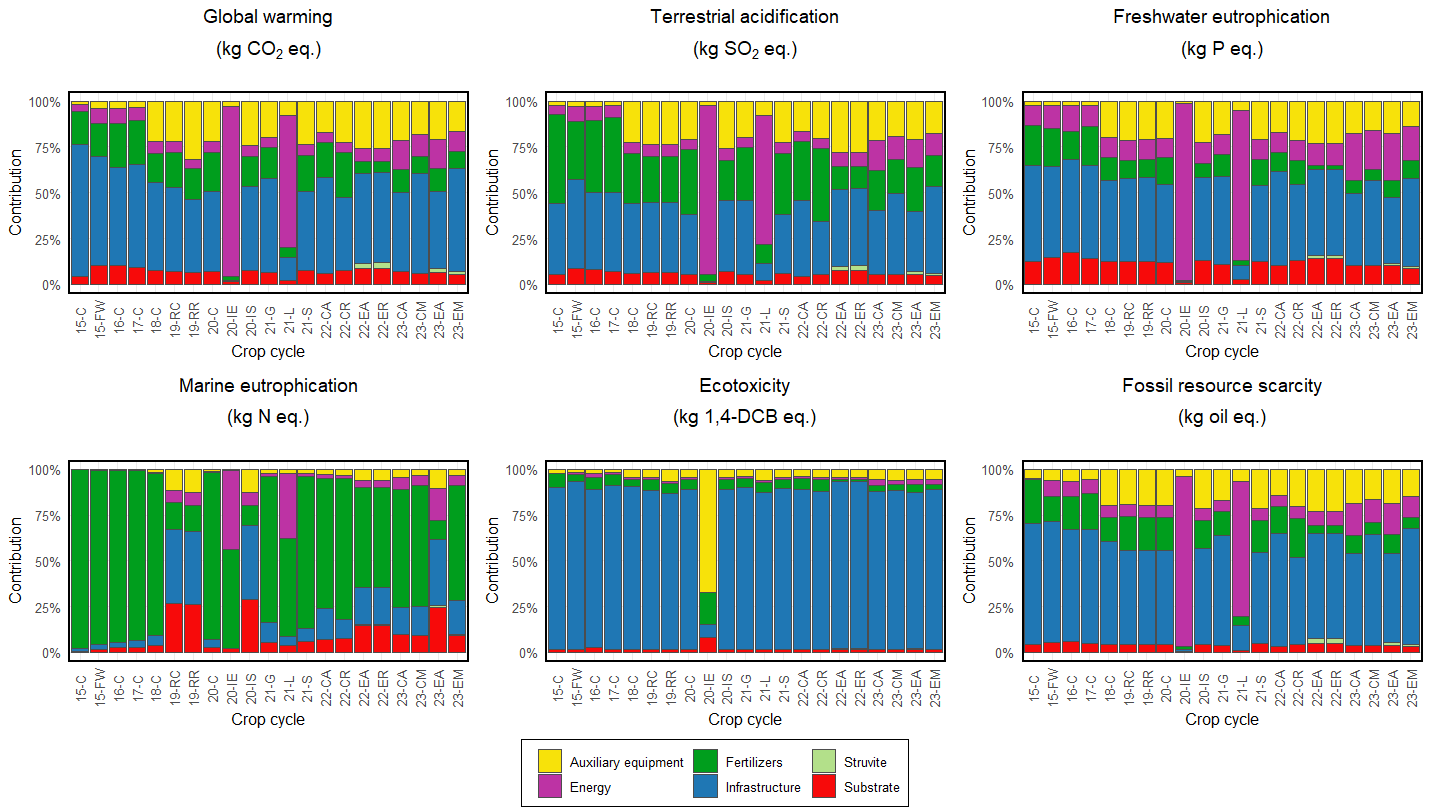


Fig. SM4. Impact contribution of tomato cultivation between 2015 and 2023.
FW: fall-winter cycle; RC: recirculation system; RR: recirculation system and irrigation reduction; IM: irrigation module; IE: indoor environment; L: supplemental lighting sector; G: Gigawak cv.; CA: mineral fertilization and Arawak cv.; CR: mineral fertilization and Rosa de Cadiz cv.; ER: struvite fertilization and Rosa de Cadiz cv.; CM: mineral fertilization and Montgrí cv; EA: struvite fertilization and Arawak cv; EM: struvite fertilization and Montgrí cv.
The exact amount of the impacts can be consulted in the Appendix 2.

**Appendix 1F. Methodology and results of sensitivity analysis on the greenhouse structure**

Table SM3. Data of the simulated scenarios for polycarbonate substitution.

|  | S10 10 years | S6 6 years | S5 5 years | S4 4 years | S3 3 years | S2 2 years |
| --- | --- | --- | --- | --- | --- | --- |
| Yield, max (kg m^-2^) | 20.3 | 20.3 | 20.3 | 20.3 | 20.3 | 20.3 |
| Yield, min (kg m^-2^) | 5.45 | 10.4 | 12.05 | 13.7 | 15.35 | 17 |
| Yield, avg (kg m^-2^) | 12.9 | 15.4 | 16.2 | 17.0 | 17.8 | 18.7 |
| Yield, avg (kg) | 819.5 | 977.0 | 1029.5 | 1082.1 | 1134.6 | 1187.1 |
| Transmissivity, max (first year after substitution) | 50.9% | 50.9% | 50.9% | 50.9% | 50.9% | 50.9% |
| Transmissivity, min (last year before substitution) | 31.1% | 39.0% | 41.0% | 43.0% | 45.0% | 46.9% |
| Covering material lifespan | 10 years | 6 years | 5 years | 4 years | 3 years | 2 years |
| i-RTG Structure lifespan | 50 years | 50 years | 50 years | 50 years | 50 years | 50 years |

Table SM4. Environmental impact of various scenarios of the sensitivity analysis.

| Impact category | Unit | S10 10 years | S6 6 years | S5 5 years | S4 4 years | S3 3 years | S2 2 years |
| --- | --- | --- | --- | --- | --- | --- | --- |
| Global warming | kg CO_2_ eq. | 1.04 | 0.96 | 0.96 | 0.97 | 1.04 | 1.20 |
| Terrestrial acidification | kg SO_2_ eq. | 4.35E-03 | 3.93E-03 | 3.87E-03 | 3.89E-03 | 4.06E-03 | 4.55E-03 |
| Freshwater eutrophication | kg P eq. | 6.02E-04 | 5.52E-04 | 5.48E-04 | 5.57E-04 | 5.89E-04 | 6.77E-04 |
| Marine eutrophication | kg N eq. | 8.11E-04 | 6.84E-04 | 6.51E-04 | 6.22E-04 | 5.98E-04 | 5.80E-04 |
| Ecotoxicity | kg 1,4-DCB eq. | 2.12E+00 | 3.78E+00 | 4.59E+00 | 5.87E+00 | 7.99E+00 | 1.25E+01 |
| Fossil resource scarcity | kg oil eq. | 4.60E+01 | 3.86E+01 | 3.66E+01 | 3.49E+01 | 3.32E+01 | 3.19E+01 |
